# Modernising fish and shark growth curves with Bayesian length-at-age models

**DOI:** 10.1371/journal.pone.0246734

**Published:** 2021-02-08

**Authors:** Jonathan J. Smart, Gretchen L. Grammer

**Affiliations:** 1 SARDI Aquatic Sciences, West Beach, SA, Australia; 2 School of Biological Sciences, The University of Adelaide, Adelaide, South Australia, Australia; Institut de Recherche pour le Developpement, FRANCE

## Abstract

Growth modelling is a fundamental component of fisheries assessments but is often hindered by poor quality data from biased sampling. Several methods have attempted to account for sample bias in growth analyses. However, in many cases this bias is not overcome, especially when large individuals are under-sampled. In growth models, two key parameters have a direct biological interpretation: *L*_*0*_, which should correspond to length-at-birth and *L*_*∞*_, which should approximate the average length of full-grown individuals. Here, we present an approach of fitting Bayesian growth models using Markov Chain Monte Carlo (MCMC), with informative priors on these parameters to improve the biological plausibility of growth estimates. A generalised framework is provided in an R package ‘BayesGrowth’, which removes the hurdle of programming an MCMC model for new users. Four case studies representing different sampling scenarios as well as three simulations with different selectivity functions were used to compare this Bayesian framework to standard frequentist growth models. The Bayesian models either outperformed or matched the results of frequentist growth models in all examples, demonstrating the broad benefits offered by this approach. This study highlights the impact that Bayesian models could provide in age and growth studies if applied more routinely rather than being limited to only complex or sophisticated applications.

## Introduction

Understanding the growth of aquatic taxa such as fish, sharks, molluscs and crustaceans is imperative for effective fisheries assessments. Specifically, information on growth is used to assess species productivity and define population structure within integrated stock assessment models [1]. Growth information is typically ascertained through length-at-age analysis using models such as a von Bertalanffy growth model [VBGM; 2, 3]. However, biased sampling often hinders growth estimation when not all length or age classes can be effectively sampled [4]. In this situation, additional methods are often applied to account for imperfect data such as constraining model fits or interpolating data through methods such as back-calculation [5, 6]. While these can be effective, in many instances biologically implausible growth estimates can still occur to varying degrees [4].

Length-at-age models are most commonly fit using frequentist approaches such as non-linear estimation [1, 7]. When appropriate length-at-age samples are available, these methods perform adequately and produce the necessary information to inform further population analyses. However, there are often instances where unrepresentative samples compromise growth estimates. For example, large species of sharks (>3 m total length) often have biased samples as fishing gear is size selective and often cannot catch the largest or smallest size classes [8, 9]. These individuals are the most influential to growth estimation as they essentially anchor the ends of the curve at minimum and maximum sizes [1]. Age zero individuals help define the length-at-birth parameter (*L*_*0*_) while the largest individuals strongly influence estimates of asymptotic length (*L*_*∞*_). When omitted, resulting growth curves may adequately describe the length-at-age over the length and age ranges available but may provide parameter estimates that do not appropriately describe the growth of the species [9–11].

Bayesian approaches are an effective tool when frequentist approaches cannot determine appropriate estimates from the data alone. Bayesian methods incorporate prior knowledge into an analysis and produce a combined output using priors along with the data available [12, 13]. The combination of priors and model likelihoods produces posterior distributions for each parameter, which represent the inclusion of additional information in the model. When estimating species growth, prior information can be easily incorporated into length-at-age modelling by creating informative priors for *L*_*0*_ and *L*_*∞*_ based on known length-at-birth (often close to zero for bony fishes) and the species maximum length, respectively. The inclusion of informative priors in Markov Chain Monte Carlo (MCMC) analyses and have proved effective in growth analyses where they have been applied [14–18]. However, of Bayesian approaches for growth estimation are most commonly applied to more complex analyses or datasets such as hierarchical modelling [19, 20], tag recapture data [21] or length-frequency data [22]. It is reasonably rare for Bayesian approaches to be applied to standard length-at-age analyses, despite their successful applications and suitability [14, 23].

The broader use of Bayesian methods in length-at-age analyses by incorporating priors on maximum and minimum lengths could prove to be a valuable advancement in growth modelling. Length-at-birth and maximum length strongly relate to two of the three estimated parameters in growth analyses and are usually used as a cursory check for model appropriateness following an analysis. Their formal inclusion in an analysis is therefore very sensible, hence why it is surprising that the use of Bayesian methods have not flourished. This has likely not occurred for two reasons: 1) there is lack of awareness of how Bayesian models could benefit length-at-age analyses by overcoming basic and common challenges with sampling; and 2) Bayesian analyses are more complicated than frequentist approaches, requiring greater knowledge of statistical distributions, modelling processes, and specialised software [12] which means they are only applied when sophisticated methods are essential. These issues mean that Bayesian methods are often not considered when in fact they could provide an elegant solution to fully considering a species’ biology in any growth analysis.

Here, we demonstrate the effectiveness of Bayesian methods for addressing sampling issues in length-at-age analyses and their benefits. In doing so, we fully document a generalised framework that is an extension of the multi-model paradigm; where multiple models are applied and the best fitting model is selected [24–26]. This framework can be applied to any length-at-age analysis that would normally be undertaken in a frequentist approach. We contend that this approach could be used more regularly to estimate growth and aim to facilitate this by providing an R package ‘BayesGrowth’ [27] that makes this analysis more accessible to a general audience. This package is available at https://github.com/jonathansmart/BayesGrowth.

## Materials and methods

### Description of generalised Bayesian growth modelling framework

#### Multi-model paradigm for growth modelling

A contemporary modelling approach rather than the *a priori* use of the VBGM is to fit multiple growth models to the data and compare their fits using Akaike’s Information Criterion (*AIC*) [25, 28]. Multi-model approaches are preferred as they reduce the chance of fitting a model that is not well suited to the data. Three growth models are commonly applied to aquatic taxa using a multi-model approach [26]: the von Bertalanffy growth model,

$$L_{a}=L_{\infty }-(L_{\infty }-L_{0})e^{-ka},$$

the Gompertz growth model,

$$L_{a}=L_{0}e^{(\log(}{}^{L_{\infty }/L_{0})(1-e^{-ka}))},$$

and the Logistic growth model,

$$L_{a}=(L_{\infty }L_{0}e^{ka})/(L_{\infty }+L_{0}(e^{ka}-1)).$$

where *L*_*a*_ is the length-at-age *a*, *L*_*0*_ is the length-at-birth, *k* is the growth completion parameter and *L*_*∞*_ is the asymptotic length. Note that the *k* parameter is unique to each model and cannot be compared between them [26]. However, *L*_*0*_ and *L*_*∞*_ have identical interpretations between models, and in a multi-model paradigm, it is sensible to use *L*_*0*_ for the von Bertalanffy model rather than *t*_*0*_ (as is common for bony fish species) to directly compare candidate model parameters.

A multi-model approach culminates in model selection via *AIC* which is calculated as *AIC = nlog*(σ^2^) + 2*k*, *k* is the total number of parameters +1 for variance (σ^2^) and *n* is the sample size. The model with the lowest *AIC* value (*AIC*_*min*_) has the best fit to the data and is the most appropriate of the candidate models. Remaining models can be ranked using the *AIC* difference (*∆AIC*) which can be calculated for each model (*i* = 1–3) as Δ*AIC* = *AIC*_*i*_−*AIC*_*min*_. Models with *∆AIC* of 0–2 have the highest support while models with *∆AIC* of 2–10 have considerably less support and models with *∆AIC* of >10 have little or no support [29]. AIC differences can be used to calculate AIC weights (AICw) which represent the probability of choosing the correct model from the candidates:

$${AICw}_{i}=\frac{exp(-\frac{\Delta_{i}}{2})}{\sum_{j=1}^{3}exp(-\frac{\Delta_{i}}{2})}$$

In this study, we extend this multi-model paradigm beyond its this application into a generalised Bayesian framework that can be applied equivalently to this frequentist approach. This framework includes the same models, produces equivalent and interchangeable outputs, and culminates in model selection. Additionally, this generalised Bayesian framework explicitly includes valuable biological information as Bayesian priors.

#### Generalised framework for MCMC growth estimation

Extending the multi-model paradigm into a Bayesian approach requires the introduction prior information for the growth parameters (*k*, *L*_*0*_ and *L*_*∞*_). Each parameter requires a prior, which can take one of several different distributions. Length-at-age models are most often fit using multi-variate normal distributions. However, each prior does not need to have the same distribution as its posterior distribution. In this study and the ‘BayesGrowth’ R package, *L*_*0*_ and *L*_*∞*_ have normally distributed priors determined from reported length-at-births and maximum lengths, respectively. The remaining growth parameter (*k*) has a uniform distribution bounded from zero to a maximum probable value. The use of a uniform distribution produces a “non-informative prior” where no additional information is provided for this parameter. Therefore, in this model, prior knowledge of *L*_*0*_ and *L*_*∞*_ is being included while no prior knowledge is provided for *k*. This allows the model to fit closely to the species length-at-birth and maximum length while permitting *k* to fluctuate and produce the necessary curvature. A fourth model parameter, the residual standard error (*σ*) also requires a prior, which is also uniform. All model priors are bounded at zero as they must remain positive. However, this means that the prior for *L*_*0*_ will become a half-normal distribution when this parameter approaches zero (as will likely occur for teleost fishes). However, for species with lengths-at-birth that are far greater than zero, this prior distribution will automatically become normally distributed. Note that as *L*_*0*_ has a direct biological meaning, it is easier to determine a prior for than *t*_*0*_.

This Bayesian structure is well suited to a multi-model paradigm for several reasons. Firstly, *L*_*0*_, σ and *L*_*∞*_ have consistent interpretations between candidate growth models and therefore the same priors can be used across models. Secondly, as the growth completion parameter (*k*) is different for each candidate model, the use of an uninformative prior again means that all three candidate models can be specified with identical priors. However, it should be noted that the upper bound for the priors of *k* and σ used in this framework should be set at values well above the expected estimates of these parameters in each candidate model. For example, an expected estimate of *k* for the VBGF may be 0.3 yr^-1^ and therefore a prior of *U(0*, *0*.*5)* would seem sensible. However, this would not be the case if the expected estimate of *k* for the logistic mode was 0.7 yr^-1^ as the prior distribution does not extend to this value. In this example setting a prior of *U(0*, *0*.*5)* would constrain the fit of the logistic model. Therefore, if consistent priors are to be used across candidate models in this framework then the upper bound of any uniform distribution must suit all models. Setting a cautiously high upper bound is sensible as this won’t influence the model and will have little effect on how quickly a model converges. This consistency in candidate model structure means that model selection is not reliant on the individual model specifications but rather on which model shape best suits the application. This significantly simplifies the use of multi-model approach in Bayesian growth model fitting.

The main outputs of Bayesian models are the posterior distributions of each model parameter. The posterior distribution is determined from a prior distribution and a likelihood distribution using Bayes’ theorem:

$$\mathrm{Pr}(\theta |Y)=\frac{\mathrm{Pr}(Y|\theta )\mathrm{Pr}(\theta )}{\int \mathrm{Pr}(Y|\theta )\mathrm{Pr}(\theta )d(\theta )}$$

where Pr is the probability, *Y* is the length-at-age data and *θ* are the parameters (*k*, *σ*, *L*_*0*_ and *L*_*∞*_). The posterior distribution is Pr(*θ*|*Y*), the prior distribution is Pr(*θ*), the likelihood distribution is Pr(*Y*|*θ*) and ∫Pr(*Y*|*θ*)Pr(*θ*)*d*(*θ*) is a normalising constant that which ensures that the posterior distribution integrates to one.

Markov Chain Monte Carlo (MCMC) was used to apply Bayes theorem to growth estimation. MCMC is a process for sampling from a posterior distribution via Markov chains to determine parameter uncertainty from the posterior distribution. Each simulation is a random draw where each is probabilistically related to the previous iteration. The MCMC produces a vector of posterior parameter estimates, which can be summarised using a central tendency (such as the mean, median or mode), and a variance (standard deviation or percentiles). Summarising a normally distributed posterior distribution by using a mean and standard deviation is equivalent to determining a frequentist parameter estimate and standard error.

For a growth curve fit using MCMC, the posterior distributions are influenced by both the length-at-age data (likelihood distributions) and the known species length-at-birth and maximum length (prior distributions). A scenario where the likelihood distributions have narrow variance means that the growth model fits the data well. Therefore, posterior distributions will be strongly weighted towards it. However, a situation where the likelihood distributions have wide variance (due to poor fit or large residuals), posterior distributions will be more strongly influenced by the priors. In this instance, inclusion of prior knowledge of the species maximum length or length-at-birth will adjust the model towards these known values. The inclusion of additional information through priors also produces more precise length-at-age predictions. These are valuable in future analyses that rely on these estimates to convert between ages and lengths. Two circumstances are particularly well suited to Bayesian methods: when data are sparse or when data are unreliable and incomplete [12]. Length-at-age samples regularly fall into these two categories [5], hence the potential improvement that can be offered by MCMC methods.

Model selection for Bayesian models can be accomplished through several different methods. However, leave-one-out-cross-validation (LOOCV) is the most contemporary and robust method presently available [30]. LOOCV estimates pointwise out-of-sample prediction accuracy using the log-likelihood evaluated at the posterior parameter distributions. Using LOOCV, leave-one-out-information-criterion (LOOIC) can be calculated which has the same application as AIC in a frequentist setting. LOOIC weights (LOOICw) for each candidate model can also be calculated and have identical interpretation as AICw for model selection.

#### BayesGrowth R package

The implementation of MCMC growth models is more computationally demanding and complicated than frequentist growth models. Several computer programs exist that perform the MCMC computations, one of which is ‘Stan’ [31]. Stan allows users to perform MCMC using both Hamiltonian Monte Carlo (HMC) and No U-Turn Sampling (NUTS), both of which are computationally efficient. Stan also calculates LOOIC which is also computationally expensive. Stan can be used in the R programming environment [32] to build MCMC models via the ‘rstan’ R package [33] and perform LOOIC using the ‘loo’ R package [34].

The generalised growth modelling framework presented here has been encapsulated in the BayesGrowth R package [27]. This package provides a series of wrapper functions around rstan models, allowing the users to perform an MCMC growth analysis (using NUTS) much more simply. Three growth models are included in ‘BayesGrowth’: the VBGM, Gompertz and Logistic growth models. These are fit with a normal residual error structure (σ) and their parameters are directly comparable to a standard growth model fit using the ‘nls’ function in ‘R’ [32]. The BayesGrowth package will fit an MCMC model via rstan while providing necessary control to the user. A rstan model object is returned from the estimation function, which allows the user to take advantage of all the auxiliary functions such as model summary statistics and diagnostic plots that exist in the supporting R packages. The BayesGrowth package also contains functions that provide credibility intervals around the growth curve (these are analogous to bootstrapped confidence intervals), as well as functions that facilitate model selection using LOOIC as part of a multi-model paradigm. The only additional step to fit an MCMC model using BayesGrowth is to provide priors based on known length-at-birth and maximum length.

### Comparison of Bayesian and frequentist growth model applications

To demonstrate the improvements offered by Bayesian over frequentist growth models, two analyses are presented. The first compares the accuracy and precision of both approaches when using simulated datasets with various sampling complications caused by gear selectivity. The second demonstrates the application of both approaches to four case studies, each of which represents a different species life history or a common sampling problem in growth studies.

The generalised Bayesian framework described previously was used in every application using the Bayesgrowth package. In each application four MCMC chains with 10,000 simulations were used to determine parameter posterior distributions. A burn in period of 5000 simulations was used, and initially, no thinning was performed. All models were checked for convergence using the Gelman-Rubin test, and diagnostic plots produced using the ‘Bayesplot’ R package [35] were examined to check that chains had mixed, and that autocorrelation did not occur. Autocorrelation can be present as each Markov chain simulation is reliant on the previous one and are therefore not independent. Autocorrelation can be accounted for by ‘thinning’, where every nth simulation is kept and the others discarded, thus reducing autocorrelation. Simple models or univariate models often do not require this and in most situations, thinning is not advised as it throws away data and increases runtimes [36]. However, in length-at-age modelling, some autocorrelation can occur, and therefore thinning should be considered when present. Autocorrelation was checked using diagnostic plots from the ‘Bayesplot’ R package [35] and if present, thinning was implemented, and the number of iterations accordingly increased until no autocorrelation occurred. All frequentist models were fit using the AquaticLifeHistory R package [37].

#### Case studies

To demonstrate the real-world application of Bayesian growth analysis, four examples are provided for species with different life histories or sampling issues. Both a frequentist approach and a Bayesian model are fit to these data. For blue mackerel (*Scomber australasicus*), an additional model where *L*_*0*_ is fixed at zero is also included. Three models were fit for each species: VBGM, Gompertz and Logistic models with model selection performed using LOOIC. The priors for each species are provided in Table 1. In each case study, the standard errors of *L*_*∞*_ and *L*_*0*_ priors were initially set as 10% of the maximum length and length-at-birth, respectively. These were used in an initial fit and increased or reduced as needed if the initial bias in the growth model estimates remained unresolved. The MCMC diagnostic plots for each case study and the code used to produce them are presented in S1 Appendix.

**Table 1:** Bayesian and frequentist of length-at-age results for four case studies.

Species	Parameter	Prior	Best fitting MCMC Model	MCMC posterior	Best fitting nls model	nls parameter estimates (free *L*_*0*_)	nls parameter estimates (fixed *L*_*0*_)
Silvertip shark	*L*_*∞*_	N(300, 30)	VBGM	296.2 ± (22.53)	VBGM	899.4 ± (1429)	-
	*k*	*U*(0, 0.3)		0.06 ± (0.01)		0.009 ± (0.02)	-
	*L*_*0*_	*p*_*[0*, *∞]*_[*N*(68, 5)]		77.22 ± (4.26)		100.7 ± (6.4)	-
	*σ*	U(0,100)		11.71 ± (1.33)		10.28	-
Silky shark	*L*_*∞*_	*N*(280, 15)	Logistic	269 ± (5.41)	Logistic	268.3 ± (6.05)	-
	*k*	*U*(0, 5)		0.14 ± (5e-3)		0.14 ± (6e-3)	-
	*L*_*0*_	*p*_*[0*, *∞]*_[*N*(77, 5)]		82.3 ± (1.57)		82.6 ± (1.71)	-
	*σ*	U(0, 100)		14.7 ± (0.45)		14.63	-
Reef ocean perch	*L*_*∞*_	N(47, 0.5)	VBGM	46.2± (0.5)	Logistic	30.4 ± (0.4.9)	-
	*k*	*U*(0, 1)		0.07 ± (3e-3)		0.27 ± (0.03)	-
	*L*_*0*_	*p*_*[0*, *∞]*_[*N*(1, 0.1)]		1 ± (0.1)		0.84 ± (1.4)	-
	*σ*	U(0,100)		3.3 ± (0.2)		2.21	-
Blue mackerel	*L*_*∞*_	*N*(44, 5)	VBGM	31.8 ± (0.43)	VBGM (Free and fixed *L*_*0*_)	37.8± (2.37)	2.93 ± (0.13)
	*k*	*U*(0, 1)		0.66 ± (0.04)		0.15 ± (0.03)	0.99 ± (0.03)
	*L*_*0*_	*p*_*[0*, *∞]*_[*N*(0, 1e-3)]		0 ± (8e-4)		20.9 ± (0.59)	-
	*σ*	*U*(0, 100)		2.43 ± (0.088)		1.94	2.17

All lengths are total length (TL) in cm. Values in brackets are the SD and SE of MCMC and frequentist models, respectively. Model results are displayed for the best fitting models, as determined by *DIC* and *AIC* (Table 2). An nls model with a *L*_*0*_ fixed at zero was only applied for Blue mackerel.

Reef ocean perch (*Helicolenus percoides*) from continental shelf-waters off the coast of South Australia were used to demonstrate the MCMC model versus frequentist growth models on a dataset lacking older fish (n = 153). These fish live their entire lives on the seafloor at depths up to 350 m, are long-lived (>40 years) and can attain lengths >40 cm [38, 39]. Ocean perch are live bearing (lecithotrophic)—most bony fish spawn eggs—and produce a gelatinous mass containing fully-developed larvae [40].

Silvertip sharks (*Carcharhinus albimarginatus*) from Papua New Guinea were used in a length-at-age analysis by Smart et al [10] from a limited sample. Silvertip sharks are born at 63–81 cm total length (TL) and can reach ~300 cm TL [41]. However, the sample in Smart et al [10] contained a length range of 95–250 cm TL from 48 sharks. Consequently, the growth estimates for these data had overestimated values of *L*_*0*_ and *L*_*∞*_. Back-calculation [42] was applied to increase the interpolated sample size and better model *L*_*0*_. However, *L*_*∞*_ was still overestimated with back-calculated data [10]. Here the observed data was used as case study to demonstrate the improvements offered by Bayesian models for both the *L*_*0*_ and *L*_*∞*_ parameters.

Silky sharks (*Carcharhinus falciformis*) from Papua New Guinea were used in length-at-age analysis by Grant et al [43] from a sample of n = 553. *Silky sharks* are born at 56–87 cm total length (TL) and can reach ~330 cm TL [41]. Grant et al [43] were able to produce a biologically plausible growth curve from these data, which corresponded to length-at-birth and resembled maximum length. These data are included as an example to compare a Bayesian model to a well performing frequentist growth model

Lastly, Blue mackerel *(Scomber australasicus)* were collected off the southern coast of New South Wales, Australia (n = 789). Blue mackerel are a small pelagic fish (forage fish) found throughout the Pacific Ocean in coastal and continental shelf waters. Younger fish usually live in inshore waters, while larger adults form schools in deeper waters (40–200 m) across the continental shelf [44]. Blue Mackerel reach sizes of up to 44 cm and at least 8 years in the Great Australian Bight [45, 46]. This dataset illustrates the fit of the Bayesian model to data for a fast-growing species where smaller fish are under-represented.

#### Selectivity scenario simulations

To compare the performance of Bayesian and frequentist growth models, three length-at-age datasets for scenarios with different selectivity functions were simulated from a VBGM. The results of the Bayesian and frequentist models were then compared to the known values used to simulate these data. The length-at-age data were drawn from a VBGM with a multiplicative error structure:

$$L_{a}∼N({\bar{L}}_{a},{\bar{L}}_{a}{}^{\sigma }),$$

$${\bar{L}}_{a}=L_{\infty }-(L_{\infty }-L_{0})e^{-ka}$$

where ${\bar{L}}_{a}$ is the mean simulated length-at-age *a* and ${\bar{L}}_{a}{}^{\sigma }$ is the multiplicative error of length *a*. The VBGM parameters used to simulate these data were *L*_*∞*_ = 250, *k* = 0.2yr^-1^, *L*_*0*_ = 0 and *σ* = 0.5. Using a multiplicative error structure represents the increasing individual variation in growth that occurs with age.

The probability of sampling different ages in each scenario was dependent on the selectivity-at-age *a* (*S*_*a*_) and the survivorship-to-age *a* (*l*_*a*_). Survivorship-to-age *a* is important as relatively fewer older individuals are present in a population as the probability of survival decreases with age. Therefore, the probability of sampling any given *L*_*a*_ was expressed using a multinomial observation process where the probability of sampling an individual of age *a* was:

$$P(L_{a})∼Mult(1,l_{a}*S_{a})$$

where the survivorship-to-age *a* (*l*_*a*_) was calculated in each scenario using a natural mortality (*M*) of 0.2 yr^-1^ as

$$l_{a}=l_{a}-1*e^{-M}.$$

Three selectivity functions were used in each scenario:

1) a logistic function where selectivity increases with age,

$$S_{a}=1/(1+e^{-slope*(a-a_{50})})$$

where the slope was 0.7 and the age-at-50%-maturity (*a*_50_) was 12 years,

2) a logistic function where selectivity decreases with age,

$$S_{a}=1/(1+e^{slope*(a-a_{50})}),$$

where the slope was 1 and the age-at-50%-maturity (*a*_50_) was 1.5 years, and 3) a dome shaped selectivity which was normally distributed with a mean age of 10 years and a standard deviation of 2 years,

$$S_{a}\sim N(10,2)$$

The code to reproduce this analysis is provided in S2 Appendix.

### Choice of informative priors

As the priors for *L*_*0*_ and *L*_*∞*_ are normally distributed, they require a standard error to be specified which will affect how wide or narrow the prior distributions are. As a more precise prior will provide greater weight to the posterior this means that the choice of standard error can be influential. Particularly, this will occur when sample sizes are low as the precision of the likelihood component of the Bayesian model will be lower. To demonstrate the effect of prior precision for *L*_*0*_ and *L*_*∞*_ on Bayesian growth models; two of the case studies (Silvertip sharks and silky sharks) were examined further. These two examples were chosen as they have large lengths-at-birth and therefore the *L*_*0*_ parameters will be greater than zero and the standard errors of the priors will be more influential. In these examples, the Bayesian models were fit three times using the original priors (base case priors) and priors with less precision that were set at 25% and 50% of the mean value used for each prior. The silvertip shark example demonstrates the effect prior precision with a low sample size (n = 48) while the silky shark example demonstrates this for a larger sample size (n = 553). The code to reproduce is provided in S3 Appendix.

## Results

### Case studies

**Fig 1:**
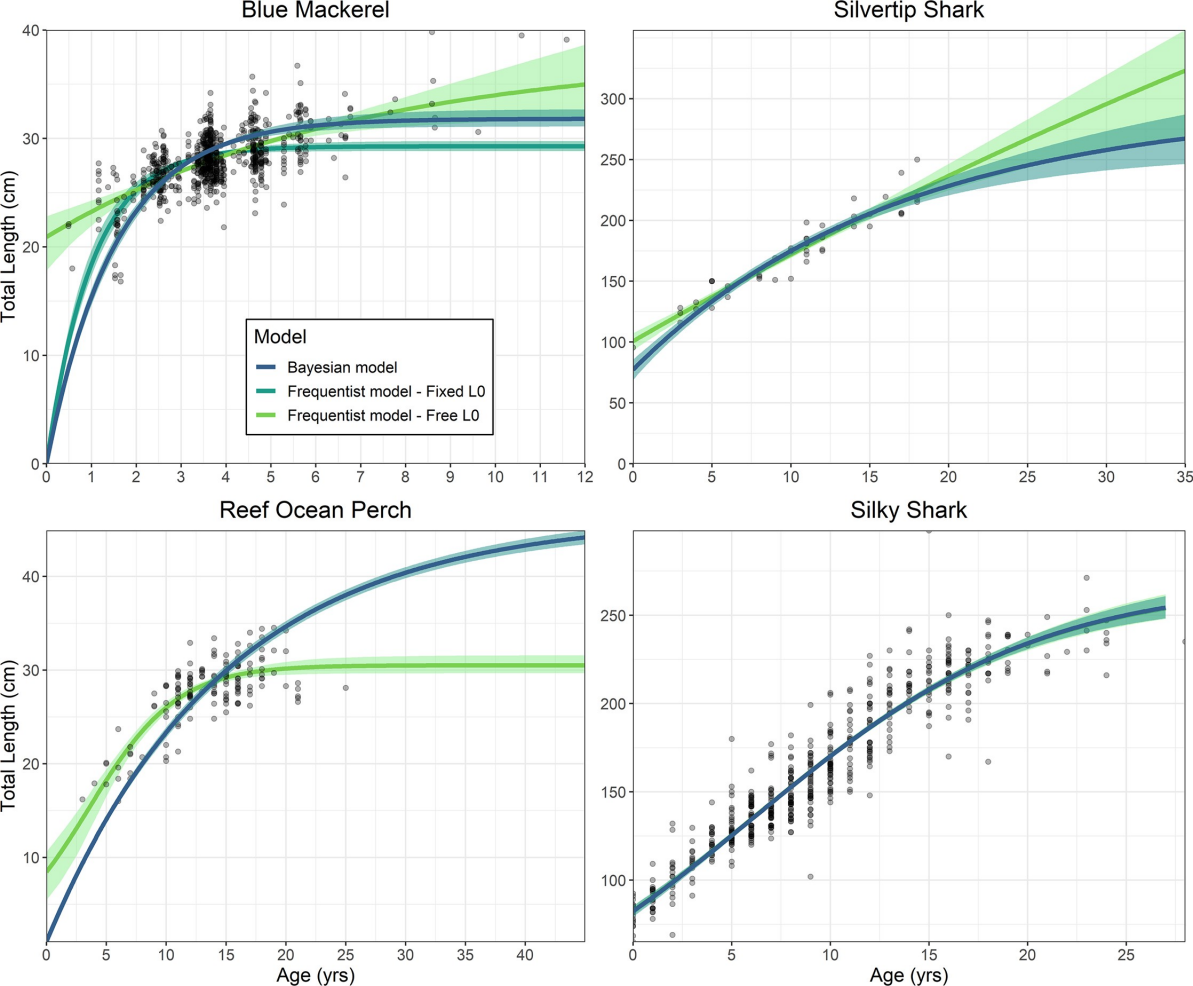
A comparison between length-at-age estimates determined from an MCMC analysis performed using Bayesian and frequentist models for the four case studies: Blue mackerel, ocean perch, silvertip shark and silky shark. All four case studies had a frequentist model with a freely estimated *L*_*0*_ included. Additionally, blue mackerel had a frequentist model with an *L*_*0*_ fixed at zero. Shaded areas correspond to 95% credibility intervals for the Bayesian length-at-age estimates and bootstrapped confidence 95% intervals for the frequentist models.

The Bayesian analysis improved the length-at-age results for silvertip sharks, blue mackerel and ocean perch, while returning negligible differences to length-at-age estimates from the frequentist model for silky sharks (Fig 1). The Bayesian model for silvertip sharks produced similar length-at-age results to the frequentist model between ages 3 and 14, after which the Bayesian model asymptoted sooner (Fig 1). This provided a more appropriate *L*_*∞*_ that corresponded to the species biology Similarly, the *L*_*0*_ was estimated lower for the Bayesian model which more closely matches the known length-at-birth.

Blue mackerel had greatly improved fits provided by the MCMC model, which fit well to the length-at-age data and had an *L*_*0*_ and *L*_*∞*_ that better matched known length-at-birth and maximum length (Table 1). The two frequentist models provided poor fits as neither produced biologically appropriate parameters. The model with a fixed *L*_*0*_ underestimated *L*_*∞*_ while, the model with a freely estimated *L*_*0*_ overestimated this parameter and *L*_*∞*_ (Fig 1).

The frequentist model for ocean perch initially appears to fit the data well (Fig 1). However, prior knowledge that this species can reach 42 years of age and 47 cm TL demonstrates that the absence of older and large fish has compromised these estimates. The Bayesian model was able to overcome this by producing a growth curve with appropriate length-at-age estimates and *L*_*∞*_ (Fig 1). A VBGM was selected by LOOIC which differed to a Logistic model that performed better in the frequentist analysis (Table 2).

**Table 2:** *LOOIC* and *AIC* results of Bayesian and frequentist length-at-age analyses for each case study.

Species	Model	*LOOIC*	*LOOICw*	*AIC* (Free *L*_*0*_)	*AICw* (Free *L*_*0*_)	*AIC* (fixed *L*_*0*_)
Silky shark	VBGM	4357	0	4348	0	-
	Logistic	**4322**	**1**	**4321**	**1**	-
	Gompertz	4333	0	4332	0	-
Silvertip shark	VBGM	**374**	**0.69**	**365.8**	**0.36**	-
	Logistic	378	0.09	366.1	0.31	-
	Gompertz	376	0.22	365.9	0.33	-
Reef ocean perch	VBGM	**1516**	**1**	1392.01	0.05	-
	Logistic	1799	0	**1386.69**	0.72	-
	Gompertz	1679	0	1388.98	0.23	-
Blue mackerel	VBGM	**7282**	**1**	**6925.11**	**0.69**	**7102.73**
	Logistic	14946	0	6929.41	0.08	Did not converge
	Gompertz	14620	0	6927.28	0.23	Did not converge

Best fitting models identified in bold typeface.

The Bayesian model and frequentist model for silky sharks produced only marginally different results to one another (Fig 1; Table 1). The best fitting model for both frequentist and Bayesian methods was the Logistic model, as determined by *AIC* and *LOOIC*, respectively (Table 2). Therefore, a Bayesian model produces effectively the same results as a frequentist model when a sufficient sample is available to estimate growth without need for S1–S3 Appendixes.

### Simulated length-at-age comparisons

**Fig 2:**
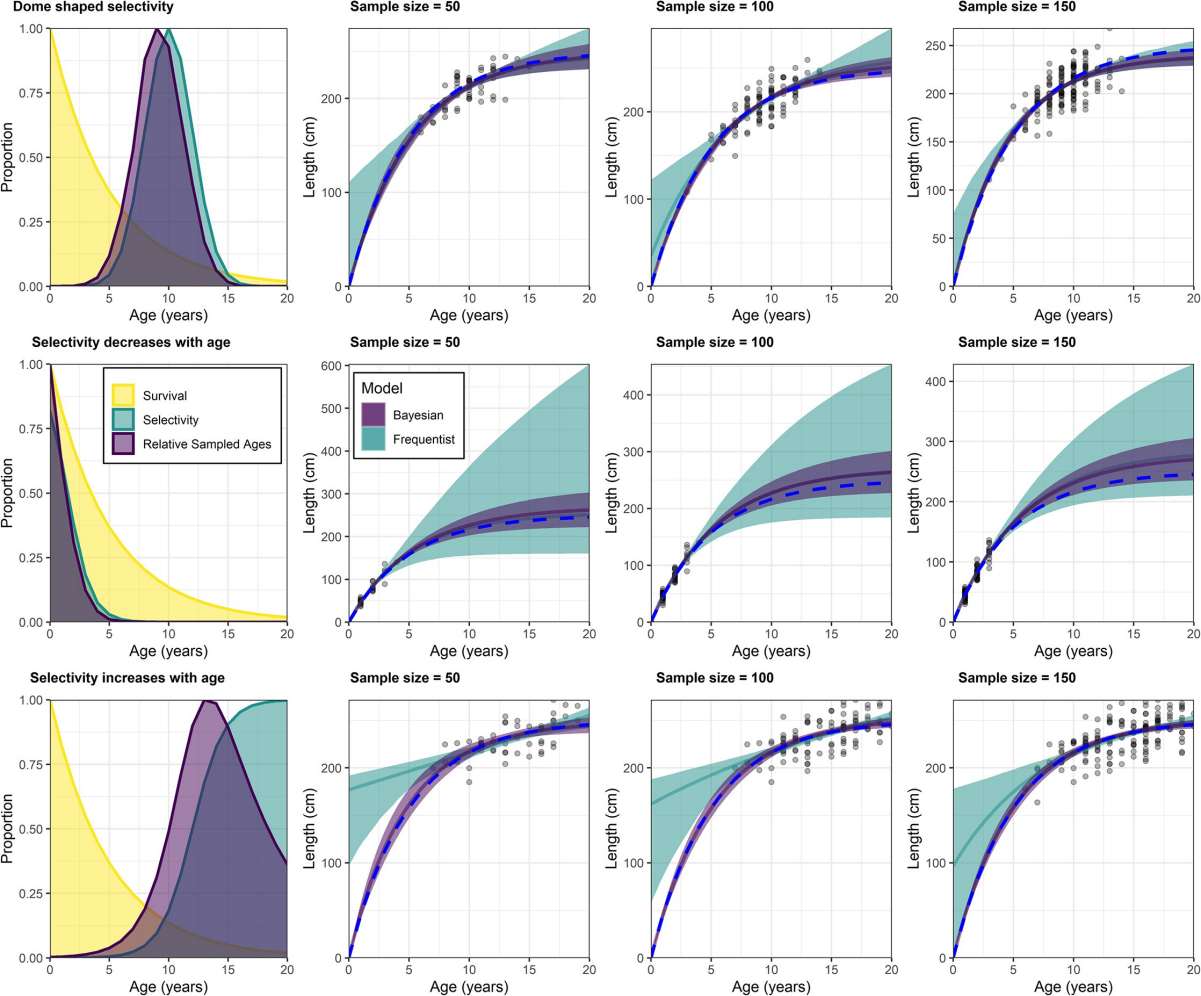
**Accuracy and precision of Bayesian (purple) and frequentist (green) growth models when fit to simulated data drawn from different selectivity functions and sample sizes (n = 50, n = 100 and n = 150).** Growth models with different sample sizes are shown in each row for the corresponding selectivity curves with relative sampled ages (product of selectivity and survivorship at age) in the left-hand panels. Shaded areas around the growth curves are the 95% bootstrapped confidence intervals for the frequentist models (green shading) and the 95% Bayesian credibility intervals (purple shading) for the Bayesian growth models. The true growth curve used to generate the length-at-age data is represented by the dashed blue line.

The Bayesian framework provided more accurate and precise length-at-age and growth parameters in eight out of nine simulations (Table 3; Fig 2). The frequentist model in the dome-shaped selectivity scenarios provided an *L*_*∞*_ that was similar to the actual *L*_*∞*_ value but had large imprecision (Table 3). However, the *L*_*0*_ parameter was overestimated with very little precision (Table 3; Fig 2). Consequently, *k* was also underestimated and imprecise (Table 3). Conversely, The Bayesian model provided good precision for all three parameters with accurate estimates of *k* and *L*_*0*_ and a reasonable estimated of *L*_*∞*_ (Table 3). Therefore, the resulting growth curve closely matched the actual growth curve that the data was drawn from (Fig 2).

**Table 3:** Comparison of parameter estimates produced by frequentist and Bayesian models using simulated length-at-age data from known parameters under different selectivity scenarios and sample sizes.

				Frequentist estimate (SE)			Bayesian estimate (SE)		
Scenario	Parameter	Known value	Priors	n = 50	n = 100	n = 150	n = 50	n = 100	n = 150
Dome based selectivity	*L*_*∞*_	250	*N*(275, 27.5)	246.9 (15.15)	268.4 (21.16)	239 (9.0)	249.59 (9.34)	257.31 (7.82)	240.3 (5.27)
	*k*	0.2	*U*(1e-5, 0.5)	0.20 (0.07)	0.15 (0.05)	0.22 (0.05)	0.19 (0.02)	0.19 (0.01)	0.22 (0.02)
	*L*_*0*_	0	*p*_*[0*, *∞]*_*[N*(0, 1e-3)]	0.01 (91.68)	34.76 (37.3)	0.01 (44.0)	0.01 (0.01)	0.01 (0.01)	0.01 (0.01
Selectivity decreases with age (Logistic)	*L*_*∞*_	250	*N*(275, 27.5)	255.6 (49.1)	269.6 (40.41)	285.9 (38.82)	269.01 (24.22)	271.4 (22.38)	278.2 (21.3)
	*k*	0.2	*U*(1e-5, 0.5)	0.20 (0.05)	0.18 (0.04)	0.17 (0.02)	0.19 (0.02)	0.18 (0.02)	0.18 (0.02)
	*L*_*0*_	0	*p*_*[0*, *∞]*_*[N*(0, 1e-3)]	0.16 (1.39)	0.18 (0.97)	0.08 (0.8)	0.01 (0.01	0.01 (0.01	0.01 (0.01
Selectivity increases with age (Logistic)	*L*_*∞*_	250	*N*(275, 27.5)	286490 (1.838e+09)	321.5 (162)	261 (13.62	248.68 (6.13)	252.63 (4.26)	250.57 (3.5)
	*k*	0.2	*U*(1e-5, 0.5)	0.00001 (0.088)	0.04 (0.08)	0.13 (0.06)	0.21 (0.03)	0.2 (0.02)	0.2 (0.01)
	*L*_*0*_	0	*p*_*[0*, *∞]*_*[N*(0, 1e-3)]	176.8 (29.65)	161.8 (39.8)	96.79 (55.24)	0.01 (1.65)	0.01 (0.01)	0.01 (0.01)

The scenarios where selectivity decreases with age via a logistic function represents a gear selectivity that targets younger individuals. In this scenario the frequentist estimates of *k* and *L*_*0*_ were accurate and precise, while *L*_*∞*_ had low precision (Table 3). The Bayesian estimates were also more accurate and precise in these scenarios for each parameter, with the only exception being the simulation with a sample size of 50 (Table 3). In this scenario, the frequentist estimates were more accurate. However, they also had unreasonably large imprecision, especially when bootstrapped confidence intervals were calculated (Fig 2). The Bayesian growth curves matched the actual growth curve very closely with good precision (Fig 2). However, *L*_*∞*_ remained overestimated in comparison to the other selectivity scenarios. This was caused by the *L*_*∞*_ prior of 275 cm which was used to represent that maximum length is often larger than *L*_*∞*_. Subsequently, the posterior of *L*_*∞*_ in this scenario was strongly weighted toward this prior (Table 3).

The scenarios where selectivity increased with age via a logistic function represents gear selectivity that targets older individuals while excluding juveniles. The frequentist model for these scenarios provided precise and accurate estimate of *L*_*∞*_ but an overestimate of *L*_*0*_ for all three sample sizes (Table 3). Therefore, *k* was underestimated (Table 3). The estimates of *L*_*0*_ also had a very low level of precision, although this didn’t affect length-at-age estimates for older age classes (Table 3; Fig 2). The Bayesian model for these scenarios provided precise parameter estimates that closely matched the actual growth parameters (Table 3). The resulting growth curve had a high level of accuracy and precision (Fig 2).

The Bayesian models were less influenced by sample size in each selectivity scenario when compared with the frequentist models (Fig 2). Posterior precision increased with sample size in each scenario while there was little change in the overall accuracy of each growth curve (Table 3; Fig 2). Conversely, the frequentist models were more strongly influenced by sample size, particularly in the scenario where selectivity increased with age. Here the accuracy of *L*_*0*_ was particularly influenced by sample size.

A *L*_*∞*_ prior that was intended to represent a maximum length that was larger than the actual *L*_*∞*_ was appropriate for the dome-shaped selectivity scenario and the scenarios where selectivity increased with age (Table 3; Fig 2). Here, consistent model estimates were produced regardless of sample size. In the scenario where selectivity decreased with age, it was apparent that this prior was more influential and caused some overestimation of *L*_*∞*_ regardless of the sample size (Table 3; Fig 2). This highlights that when older individuals cannot be sampled, particular care should be placed on selecting a prior for *L*_*∞*_.

### Choice of informative priors

**Fig 3:**
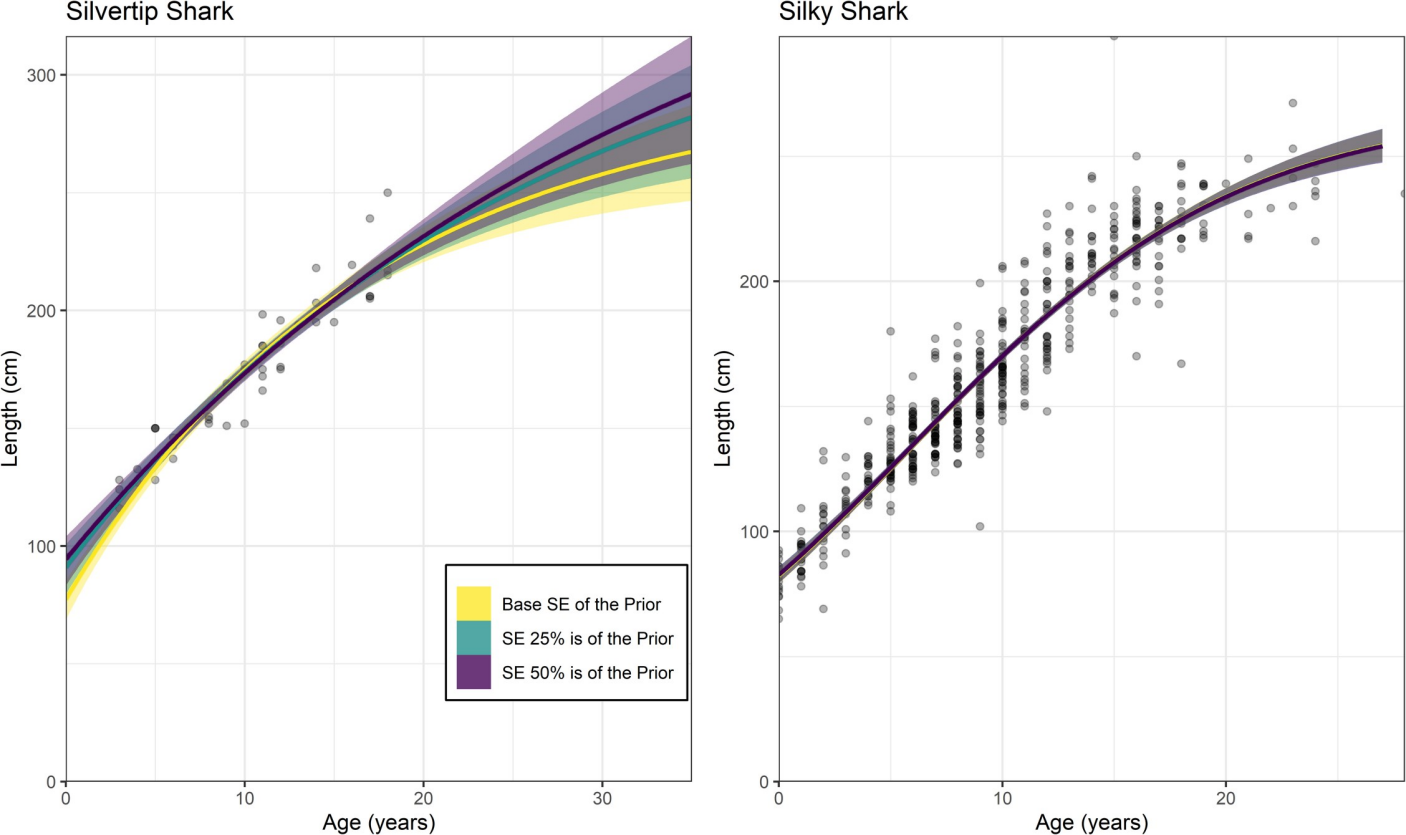
A comparison of Bayesian growth models fit to silvertip and silky sharks using different levels of precision on the *L*_*0*_ and *L*_*∞*_ priors. The base case scenarios (yellow) represent the priors used in each case study (Table1). The other scenarios include priors where the standard error (SE) is 25% of the value used in each prior (green) and 50% of the value used in each prior (purple).

Altering the precision of *L*_*0*_ and *L*_*∞*_ priors only affected the silvertip shark application where the sample size was n = 48 (Table 4; Fig 3). The silky shark application which had a much larger sample size (n = 553), had only negligible changes to its posteriors and resulting growth curve (Table 4; Fig 3). The posteriors for all parameters changed according to the priors used for silvertip sharks with *L*_*0*_ and *L*_*∞*_ better matching the known maximum size and length-at-birth when the priors were more precise (Table 4). Additionally, the standard error of the with *L*_*0*_ and *L*_*∞*_ had much greater precision when a narrower prior was used (Table 4). The greatest amount of variation occurred for with *L*_*∞*_ which increased by approximately 40% when a weakly informative prior used rather than the base case prior (Table 4). However, the results of all of these Bayesian models provide more precise parameter estimates than the corresponding frequentist models for silvertip sharks (Tables 1; 4).

**Table 4:** Comparison of prior precision for *L*_*0*_ and *L*_*∞*_ for silvertip sharks and silky sharks.

Silvertip sharks (n = 48)						
Parameter	Base case prior		SE is 25% of mean		SE is 50% of mean	
	Prior	Posterior (SE)	Prior	Posterior (SE)	Prior	Posterior (SE)
*L*_*∞*_	N(300, 30)	296.2 (22.53)	N(300, 75)	357 (47.71)	N(300, 150)	419.7 (84.32)
*k*	*U*(0, 0.3)	0.06 (0.01)	*U*(0, 0.3)	0.04 (0.01)	*U*(0, 0.3)	0.03 (0.01)
*L*_*0*_	*p*_*[0*, *∞]*_[*N*(68, 5)]	77.22 (4.26)	*p*_*[0*, *∞]*_[*N*(68, 17)]	91.13 (5.24)	*p*_*[0*, *∞]*_[*N*(68, 34)]	94.55 (5.23)
*σ*	U(0,100)	11.71 (1.33)	U(0,100)	10.79 (1.2)	U(0,100)	10.64 (1.16)
Silky Sharks (n = 553)						
*L*_*∞*_	*N*(280, 15)	269 (5.41)	*N*(280, 70)	268.7 (5.78)	*N*(280, 140)	268.7 (5.79)
*k*	*U*(0, 5)	0.14 (0.01)	*U*(0, 5)	0.14 (0.01)	*U*(0, 5)	0.14 (0.01)
*L*_*0*_	*p*_*[0*, *∞]*_[*N*(77, 5)]	82.3 (1.57)	*p*_*[0*, *∞]*_[*N*(77, 19.25)]	82.59 (1.67)	*p*_*[0*, *∞]*_[*N*(77, 38.5)]	82.58 (1.66)
*σ*	U(0, 100)	14.7 (0.45)	U(0, 100)	14.68 (0.45)	U(0, 100)	14.67 (0.45)

## Discussion

Bayesian methods have previously been applied in shark and fish growth models with great effectiveness [14, 16, 17, 23, 47]. However, these applications have been mostly limited to more complex analyses rather than typical length-at-age analyses, presenting an opportunity to broaden their use. Given that Bayesian models can formally include information on species biology, it is surprising that this technique has not become more common for growth modelling as is the case in other ecological studies [48]. The generalised framework presented here directly addresses this by providing a flexible approach that could be broadly applied to standard growth analyses and will provide improved or equivalent results to a frequentist approach.

The greatest improvement offered by this generalised Bayesian framework is the ability to account for missing older individuals caused by decreased selectivity with age. It is common for older individuals to be under sampled, particularly for large or long-lived species as the probability of surviving to older ages can be low for fished populations. While methods such as fixing *L*_*0*_ or applying back-calculation can account for the opposite scenario (missing younger individuals), to date there have been few approaches that have effectively accounted for missing older individuals. Some studies have attempted to fix *L*_*∞*_ to the known maximum size [49–51]. However, this has resulted in poor model performance when applied as part of a multi-model paradigm [51]. Here, the inclusion of a prior on maximum length effectively improved the estimate of *L*_*∞*_ when older individuals were missing in both the simulated example and the Silvertip shark case study. To date, this may be the greatest solution to under sampling of older individuals due to gear selectivity or age truncation that has hindered numerous growth studies [9–11].

While using maximum length as a prior for *L*_*∞*_ was effective in these examples, there may situations when further refinement is required. For example, *L*_*∞*_ represents the average length of fully grown individuals [7, 52] and therefore maximum length is likely to be larger than this value for many species. In situations where *L*_*∞*_ is underestimated, using maximum length as the *L*_*∞*_ prior will be beneficial as it will increase the *L*_*∞*_ posterior and account for this underestimation, as demonstrated here for Blue Mackerel. However, when *L*_*∞*_ is overestimated, using maximum length as the prior for *L*_*∞*_ will be less influential than if it were underestimated. The Silvertip example provided in this study demonstrates this, although biologically appropriate results were still produced. The simulations where selectivity decreased with age demonstrated that when older individuals were sparse, some overestimation of *L*_*∞*_ occurred when setting its prior based on a hypothetical maximum length that was 5% larger than the actual value. This demonstrates that the prior for *L*_*∞*_ requires the most consideration, especially when older individuals are under sampled in order to gain the most benefit from a Bayesian approach.

Contemporary growth modelling approaches incorporate a multi-model paradigm, where several models are fit and the best performing model is selected [24, 25]. This approach has been extended into the Bayesian space through several studies that applied multiple models [20, 53–56]. However, one complication posed by a Bayesian approach is that parameters differ between models. This requires different priors to be specified; a complication which had no previous solution [57]. Therefore, many of multi-model Bayesian applications have compared models that were similar or variants of the same model shape (i.e. VBGM). Model selection of Bayesian growth models with fundamentally different shapes is more complicated, since a candidate model that is poorly specified may not be selected, even if its shape best fits a species’ growth form. This would occur if an inappropriate prior were place on a candidate model’s growth completion parameter (*k*). In this situation the resulting posterior distribution may be heavily weighted to the prior and effect model selection. Determining priors for the growth completion parameters is more complicated as these parameters have no biological interpretation. Therefore, applying a weakly information or uninformative prior for *k* may be more appropriate to avoid miss specification. The framework presented here allows consistent priors to be applied across three different candidate models. This is achieved by ensuring that all models are parameterised to use *L*_*0*_ and *L*_*∞*_—which have informative priors—while applying uninformative priors on remaining parameters. The application of a Bayesian multi-model framework is now greatly simplified, because model specification will not affect model performance, allowing the most suitable model for the species to be selected.

The results of both analyses presented here demonstrate the improvements offered by Bayesian over frequentist approaches. Bayesian models provided the closest match to known growth parameters that were used to generate length-at-age data based on different selectivities. In each example, frequentist models tended to over or under-estimate length-at-ages depending on missing age classes that were omitted due to selectivity. Meanwhile, the inclusion of priors allowed the Bayesian models to better match those growth curves, providing more accurate and precise results. Selectivity has been previously examined through similar simulation studies with Frater and Stefansson [58] determining techniques that can account for this bias. However, these techniques required knowledge of the selectivity function which is not always known and can cause increased parameter bias if miss-specified [4]. The Bayesian framework presented here overcame these selectivity scenarios without knowledge of the selectivity function, offering a simple method that can improve growth model results without the risk of introducing additional bias.

Improved growth estimates were also produced in three out of four case studies presented here using the Bayesian framework. In each of these examples, known issues with the growth curves were overcome with previously poorly estimated *L*_*0*_ or *L*_*∞*_ now better matching biologically plausible values. The only example where no definitive improvement was produced was for Silky sharks, which had a larger sample size and included all age classes [43]. In this example, almost identical length-at-age estimates were produced by both Bayesian and frequentist approaches. One could therefore argue that if sufficient samples were available, then there is no need for a Bayesian approach, and the added complexity may introduce error. However, an opposing conclusion would be that applying a Bayesian approach can only improve growth estimates. We therefore conclude that there is little to be lost by using a Bayesian approach, and instead, much to be gained.

Several alternatives to a Bayesian approach have been applied extensively in growth studies to account for sampling limitations. Given that *L*_*0*_ and *L*_*∞*_ have biological interpretations, it has been common to fix these parameters at corresponding values. Fixing *L*_*0*_ at zero is especially common for bony fish species, which have a larval phase and are born at a size close to zero. However, growth model parameters co-vary [19], and fixing one parameter to a specific value biases the others [59]. For example, fixing *L*_*0*_ to a single value is not advised as slight inaccuracies in assigning the true length-at-birth can adversely bias the remaining parameters [59]. Some authors have also attempted to fix both *L*_*0*_ and *L*_*∞*_ while only estimating *k* [49–51]. However, this resulted in a poor model fit in each of these studies and was quickly dismissed as a candidate model in multi-model approaches. Gwinn et al [4] examined this topic in detail by simulating a population that was exploited under different fishery selectivity types and attempting to overcome biased sampling using different strategies. However, there was no overall strategy that could be applied across all sampling circumstances without biasing estimates. Finally, back-calculation has been applied to several species to determine theoretical lengths-at-age from older individuals [5, 42, 60]. This can effectively overcome sampling bias when large individuals have been sampled but smaller individuals are omitted. However, this cannot account for situations where larger individuals are under sampled, which often leads to overestimating *L*_*∞*_ [9–11]. The Bayesian approach presented here is the first technique that has addressed the under sampling of large individuals and their effect on growth estimation.

There are several advantages to the Bayesian framework we present, aside from the improved growth estimates demonstrated in these examples. It is intuitive to include known biological traits of a species as explicit components of a growth model. Maximum length and length-at-birth are regularly deemed synonymous with *L*_*0*_ and *L*_*∞*_, respectively [4, 59], and thus acknowledging this through priors is reasonable. Posterior distributions of model parameters also contain a great deal more information than point estimates and standard errors provided by frequentist models. For example, posterior distributions may reveal multiple modes, or show the probability mass to be concentrated in a certain area [12]. Convergence issues are easier to diagnose in Bayesian models as more information is returned to user. The user can use this additional information to step through the diagnostic tests outlined in this study and improve model fit.

The Bayesian approach detailed here is widely applicable, however, there are a few minor disadvantages to discuss. Firstly, there are more diagnostics to examine for Bayesian models than frequentist models, which can make them seem overly complex for simple analyses. However, if a model has been correctly specified, then these diagnostics add substantial confidence to the analysis. Secondly, one area where Bayesian statistics lags behind frequentist statistics is user-friendly software [12], which has likely prevented the uptake of Bayesian methods for growth modelling. The goal of our study was to directly address this with a user-friendly R package for Bayesian growth modelling. Lastly and most importantly, users of this approach need to embrace a Bayesian philosophy to model fitting. Frequentist statisticians would argue that the need to introduce priors is a weakness of Bayesian approaches and introduces subjectivity [12]. However, some degree of subjectivity is often unavoidable in any analysis, and here, the strength of Bayesian methods lies in its direct acknowledgement and justification. Including a range of sensible priors will often produce the same results as a frequentist model when data is unbiased [12], as demonstrated here in the case study for silky sharks.

Determining priors for the four case studies in the present study was not complicated. In each example, a standard error was initially set as 10% of the maximum length and length-at-birth. This was used in an initial fit and was increased or reduced as needed if the initial bias in the growth model estimates remained unresolved. The resulting growth estimates in all four instances produced growth estimates that fit the length-at-age data well and provided estimates of *L*_*0*_ and *L*_*∞*_ that corresponded to species’ biology. The same approach was used in the selectivity scenarios and produced appropriate results in each application. The model framework here does not require priors of *k* and *σ* to be determined on a case-by-case basis, as these priors are uninformative. Therefore, these priors only require an upper bound to their uniform distributions. Whether these upper bounds are appropriate is determined by whether or not their resulting posteriors are normally distributed.

In any Bayesian analysis, the user should judiciously document and explain their choice of priors, which for length-at-birth and maximum length is straightforward. However, the standard error of each of these distributions can be more complicated to determine. A narrower standard error may weigh the posterior distribution more toward the prior distribution than the likelihood distribution, although the extend of this can be dependent on sample size. For example, a wider standard error applied when sample size is large may be insufficient to update the posterior from the likelihood distribution, as seen in the present study for Silky sharks. Therefore, a narrower standard error on certain parameters may be required to ensure prior information is adequately incorporated into the model. Alternatively, when sample size is small, narrower priors can have far more influence on the posteriors, as seen in the present study for Silvertip sharks. In this example, a narrower prior on *L*_*∞*_ was justified as the initial frequentist model could not match the biology of the species. Wide priors on *L*_*∞*_ improved the results but the Bayesian model that provided the best representation of species’ biology was produced with narrower priors on *L*_*0*_ and *L*_*∞*_. In some instances, information may be available to determine a standard error for the *L*_*0*_ and *L*_*∞*_ priors. A recent example for *L*_*0*_ was performed using predicted lengths-at-age-zero from back-calculations to determine the mean and standard error of this prior [61]. Similarly, a recent study also used the lengths of the largest individuals documented in the literature to determine the prior mean and standard error for *L*_*∞*_ [62]. Justifications such as these can be important when using Bayesian models and demonstrate the opportunity for researchers to include as much biological information as possible in their analysis, thus improving the results. These considerations are the most important decisions when implementing a Bayesian growth model and can add the most benefit to the analysis.

## Conclusions

The generalised framework presented here demonstrates the impact that Bayesian methods could make for standard length-at-age studies. Previously, Bayesian has been more complicated to implement than frequentist alternatives for growth modelling, since considerably more statistical and programming expertise is needed. However, the BayesGrowth R package helps remove this barrier and introduce more users to Bayesian growth modelling. Its application in the present study to the four case studies and selectivity simulations, demonstrates the improvements offered over comparable frequentist models. Additionally, this generalised framework may be further tailored by users who seek more specified or sophisticated models. For example, some occasions may require different priors, error structures or candidate models to be applied in a similar framework. Examples already exist where biphasic or Schnute models have been used in Bayesian growth models [17, 23, 63]. Likewise, more sophisticated models can incorporate hierarchical structures [16, 19, 22, 64], identify growth morphs [54], account for autocorrelation in back calculations [65], or examine environmental drivers [64, 66]. The framework we have presented sits alongside this existing body of research by providing an entry point to Bayesian growth modelling that can be applied to standard growth modelling scenarios. With the information and guidance provided in this study, there is great opportunity for the field of Bayesian growth modelling to continue to expand and possibly become the standard approach for estimating fish growth.

## Supporting information

S1 Appendix Bayesian and frequentist analyses of case studies. (HTML)

S2 Appendix Bayesian vs frequentist growth model comparisons using simulated gear selectivities. (HTML)

S3 Appendix Model comparison using different standard errors for priors. (HTML)

S1 File (CSV)

S2 File (CSV)

S3 File (CSV)

S4 File (CSV)

## References

[1] Haddon M. Modelling and Quantitative Methods in Fisheries. 2nd ed Boca Raton, FL: CRC Press; 2012.

[2] von Bertalanffy L. A quantitative theory of organic growth (inquires on growth laws. II). Human Biology. 1938;10(2):181–213. 10.2307/41447359

[3] Beverton RJH, Holt SJ. On the Dynamics of Exploited Fish Populations. 1st ed London: Chapman and Hall; 1957.

[4] Gwinn DC, Allen MS, Rogers MW. Evaluation of procedures to reduce bias in fish growth parameter estimates resulting from size-selective sampling. Fisheries Research. 2010;105(2):75–9. 10.1016/j.fishres.2010.03.005.

[5] Smart JJ, Harry AV, Tobin AJ, Simpfendorfer CA. Overcoming the constraints of low sample sizes to produce age and growth data for rare or threatened sharks. Aquat Conserv. 2013;23(1):124–34. 10.1002/aqc.2274 WOS:000313984800011.

[6] Francis RICC. Back calculation of fish length—a critical review. Journal of Fish Biology. 1990;36(6):883–902. ISI:A1990DK64900009.

[7] Ogle DH. Introductory Fisheries Analyses in R. Boca Raton, FL: CRC Press; 2015.

[8] Harry AV, Tobin AJ, Simpfendorfer CA, Welch DJ, Mapleston A, White J, et al Evaluating catch and mitigating risk in a multispecies, tropical, inshore shark fishery within the Great Barrier Reef World Heritage Area. Marine and Freshwater Research. 2011;62(6):710–21. 10.1071/mf10155 WOS:000291995600021.

[9] Simpfendorfer CA, McAuley RB, Chidlow J, Unsworth P. Validated age and growth of the dusky shark, *Carcharhinus obscurus*, from Western Australian waters. Marine and Freshwater Research. 2002;53(2):567–73. 10.1071/mf01131 WOS:000174841500052.

[10] Smart JJ, Chin A, Baje L, Tobin AJ, Simpfendorfer CA, White WT. Life history of the silvertip shark *Carcharhinus albimarginatus* from Papua New Guinea. Coral Reefs. 2017 10.1007/s00338-016-1533-x

[11] McAuley RB, Simpfendorfer CA, Hyndes GA, Allison RR, Chidlow JA, Newman SJ, et al Validated age and growth of the sandbar shark, Carcharhinus plumbeus (Nardo 1827) in the waters off Western Australia. Environmental Biology of Fishes. 2006;77(3–4):385–400. 10.1007/s10641-006-9126-0 WOS:000242146300016.

[12] Bijak J, Bryant J. Bayesian demography 250 years after Bayes. Population Studies. 2016;70(1):1–19. 10.1080/00324728.2015.1122826 26902889 PMC4867874

[13] Link WA, Cam E, Nichols JD, Cooch EG. Of BUGS and birds: Markov chain Monte Carlo for hierarchical modeling in wildlife research. The Journal of wildlife management. 2002:277–91.

[14] Siegfried KI, Sanso B. Two Bayesian methods for estimating parameters of the von Bertalanffy growth equation. Environmental Biology of Fishes. 2006;77(3–4):301–8. 10.1007/s10641-006-9112-6 ISI:000242146300009.

[15] Vincenzi S, Mangel M, Crivelli AJ, Munch S, Skaug HJ. Determining individual variation in growth and its implication for life-history and population processes using the empirical bayes method. PLoS Computational Biology. 2014;10(9):e1003828 10.1371/journal.pcbi.1003828 25211603 PMC4161297

[16] Helser TE, Lai H-L. A Bayesian hierarchical meta-analysis of fish growth: with an example for North American largemouth bass, Micropterus salmoides. Ecological Modelling. 2004;178(3):399–416. 10.1016/j.ecolmodel.2004.02.013.

[17] Quince C, Shuter BJ, Abrams PA, Lester NP. Biphasic growth in fish II: Empirical assessment. Journal of Theoretical Biology. 2008;254(2):207–14. 10.1016/j.jtbi.2008.05.030 18606422

[18] Scherrer SR, Kobayashi DR, Weng KC, Okamoto HY, Oishi FG, Franklin EC. Estimation of growth parameters integrating tag-recapture, length-frequency, and direct aging data using likelihood and Bayesian methods for the tropical deepwater snapper Pristipomoides filamentosus in Hawaii. Fisheries Research. 2021;233:105753 10.1016/j.fishres.2020.105753

[19] Pilling GM, Kirkwood GP, Walker SG. An improved method for estimating individual growth variability in fish, and the correlation between von Bertalanffy growth parameters. Canadian Journal of Fisheries and Aquatic Sciences. 2002;59(3):424–32.

[20] He JX, Bence JR. Modeling Annual Growth Variation using a Hierarchical Bayesian Approach and the von Bertalanffy Growth Function, with Application to Lake Trout in Southern Lake Huron. 2007;136(2):318–30. 10.1577/t06-108.1

[21] Sigourney DB, Munch SB, Letcher BH. Combining a Bayesian nonparametric method with a hierarchical framework to estimate individual and temporal variation in growth. 2012;247:125–34. 10.1016/j.ecolmodel.2012.08.009

[22] Zhou S, Martin S, Fu D, Sharma R. A Bayesian hierarchical approach to estimate growth parameters from length data of narrow spread. ICES J Mar Sci. 2020;77(2):613–23. 10.1093/icesjms/fsz241

[23] Doño F, Montealegre-Quijano S, Domingo A, Kinas PG. Bayesian age and growth analysis of the shortfin mako shark Isurus oxyrinchus in the Western South Atlantic Ocean using a flexible model. Environmental Biology of Fishes. 2015;98(2):517–33. 10.1007/s10641-014-0284-1

[24] Katsanevakis S. Modelling fish growth: Model selection, multi-model inference and model selection uncertainty. Fisheries Research. 2006;81(2–3):229–35. 10.1016/j.fishres.2006.07.002 WOS:000241425000014.

[25] Katsanevakis S, Maravelias CD. Modelling fish growth: multi-model inference as a better alternative to *a priori* using von Bertalanffy equation. Fish and Fisheries. 2008;9(2):178–87. 10.1111/j.1467-2979.2008.00279.x WOS:000255703800006.

[26] Smart JJ, Chin A, Tobin AJ, Simpfendorfer CA. Multimodel approaches in shark and ray growth studies: strengths, weaknesses and the future. Fish and Fisheries. 2016 10.1111/faf.12154

[27] Smart J. BayesGrowth: Estimate fish growth using MCMC analysis. R package version 0.3.0. https://github.com/jonathansmart/BayesGrowth. 2020.

[28] Akaike H, editor Information theory as an extension of the maximum likelihood. Second International Symposium on Information Theory; 1973; Akademiai Kiado, Budapest. Akadémiai Kiado.

[29] Burnham KP, Anderson DR. Kullback-Leibler information as a basis for strong inference in ecological studies. Wildl Res. 2001;28(2):111–9. 10.1071/wr99107 WOS:000168859000001.

[30] Vehtari A, Gelman A, Gabry J. Practical Bayesian model evaluation using leave-one-out cross-validation and WAIC. Statistics and Computing. 2017;27(5):1413–32. 10.1007/s11222-016-9696-4

[31] Carpenter B, Gelman A, Hoffman MD, Lee D, Goodrich B, Betancourt M, et al Stan: A Probabilistic Programming Language. 2017;76(1). 10.18637/jss.v076.i01 PMC9788645 36568334

[32] R Core Team. R: A language and environment for statistical computing. In: Computing RFS, editor. Vienna, Austria: R Foundation Statistical Computing; 2019.

[33] Stan Development Team. RStan: the R interface to Stan. R package version 2.21.2. http://mc-stan.org/. 2020.

[34] Vehtari A, Gabry J, Magnusson M, Yao Y, Bürkner P, Paananen T, et al. loo: Efficient leave-one-out cross-validation and WAIC for Bayesian models. R package version 2.3.1, https://mc-stan.org/loo. 2020.

[35] Gabry J, T M. bayesplot: Plotting for Bayesian Models. R package version 1.7.2, https://mc-stan.org/bayesplot. 2020.

[36] Link WA, Eaton MJ. On thinning of chains in MCMC. Methods in Ecology and Evolution. 2012;3(1):112–5. 10.1111/j.2041-210X.2011.00131.x

[37] Smart J. AquaticLifeHistory: Fisheries life history analysis using contemporary methods. https://github.com/jonathansmart/AquaticLifeHistory. 10.5281/zenodo.3777743. 2019.

[38] Withell A, Wankowski J. Estimates of age and growth of ocean perch, *Helicolenus percoides* Richardson, in south-eastern Australian waters. Marine and Freshwater Research. 1988;39(4):441–57. 10.1071/MF9880441.

[39] Paul L, Horn P. Age and growth of sea perch (Helicolenus percoides) from two adjacent areas off the east coast of South Island, New Zealand. Fisheries Research. 2009;95(2–3):169–80.

[40] Pavlov D, Emel’yanova N. Transition to viviparity in the order Scorpaeniformes: brief review. Journal of ichthyology. 2013;53(1):52–69.

[41] White WT, Baje L, Sabub B, Appleyard SA, Pogonoski J, Mana R. Sharks and rays of Papua New Guinea. Canberra, Australia: ACIAR; 2018.

[42] Goldman KJ, Caillet GM, Andrews AH, Natanson LJ. Assessing the age and growth of Chondrichthyan fishes. In: Carrier JC, Musick JA, Heithaus MR, editors. Biology of sharks and their relatives: 2nd edition Boca Raton, FL, USA: CRC Press; 2012 p. 423–52.

[43] Grant MI, Smart JJ, White WT, Chin A, Baje L, Simpfendorfer CA. Life history characteristics of the silky shark Carcharhinus falciformis from the central west Pacific. Marine and Freshwater Research. 2018;69(4):562–73.

[44] Kailola P, Williams M, Stewart P, Reichlet R, McNee A, C G. Australian fisheries resources. Canberra: Bureau of Resource Sciences and Fisheries Research and Development Corporation, 1993.

[45] Stevens JD, Hausfeld HF, Davenport SR. Observations on the biology, distribution and abundance of *Trachurus declivis*, *Sardinops neopilchardus* and *Scomber australasicus* in the Great Australian Bight. Cronulla: CSIRO Marine Laboratories, 1984 Contract No.: 164.

[46] Ward T, Rogers P. Development and evaluation of egg-based stock assessment methods for blue mackerel Scomber australasicus in southern Australia. Report 2002/061. Fisheries Research and Development Corporation, Adelaide. 2007.

[47] Caltabellotta FP, Siders ZA, Murie DJ, Motta FS, Cailliet GM, Gadig OBF. Age and growth of three endemic threatened guitarfishes Pseudobatos horkelii, P. percellens and Zapteryx brevirostris in the western South Atlantic Ocean. Journal of Fish Biology. 2019;95(5):1236–48. 10.1111/jfb.14123 31429078

[48] Ellison AM. Bayesian inference in ecology. Ecology Letters. 2004;7(6):509–20. 10.1111/j.1461-0248.2004.00603.x

[49] Sammons SM, Maceina MJ. Variation in growth and survival of Bluegills and Redbreast Sunfish in Georgia rivers. North American Journal of Fisheries Management. 2009;29(1):101–8.

[50] Natanson LJ, Kohler NE, Ardizzone D, Cailliet GM, Wintner SP, Mollet HF. Validated age and growth estimates for the shortfin mako, Isurus oxyrinchus, in the North Atlantic Ocean. In: Carlson J, Goldman K, editors. Special Issue: Age and Growth of Chondrichthyan Fishes: New Methods, Techniques and Analysis. Developments in Environmental Biology of Fishes 25: Springer Netherlands; 2006 p. 367–83.

[51] Farrell ED, Mariani S, Clarke MW. Age and growth estimates for the starry smoothhound (Mustelus asterias) in the Northeast Atlantic Ocean. ICES J Mar Sci. 2010;67(5):931–9. 10.1093/icesjms/fsp295 WOS:000279870500009.

[52] Francis RICC. Are growth-parameters estimated from tagging and age length data comparable. Canadian Journal of Fisheries and Aquatic Sciences. 1988;45(6):936–42. WOS:A1988N930900002.

[53] Alos J, Palmer M, Balle S, Grau AM, Morales-Nin B. Individual growth pattern and variability in Serranus scriba: a Bayesian analysis. 2010;67(3):502–12. 10.1093/icesjms/fsp265

[54] Shertzer KW, Fieberg J, Potts JC, Burton ML. Identifying growth morphs from mixtures of size-at-age data. Fisheries Research. 2017;185:83–9. 10.1016/j.fishres.2016.09.032

[55] Ward HG, Post JR, Lester NP, Askey PJ, Godin TJCJoF, Sciences A. Empirical evidence of plasticity in life-history characteristics across climatic and fish density gradients. 2017;74(4):464–74.

[56] Yokouchi K, Daverat F, Miller MJ, Fukuda N, Sudo R, Tsukamoto K, et al Growth potential can affect timing of maturity in a long-lived semelparous fish. Biology Letters. 2018;14(7):20180269 10.1098/rsbl.2018.0269 29997187 PMC6083232

[57] Chambers MS, Sidhu LA, O’Neill B, Sibanda N. Flexible von Bertalanffy growth models incorporating Bayesian splines. Ecological Modelling. 2017;355:1–11. 10.1016/j.ecolmodel.2017.03.026

[58] Frater PN, Stefansson G. Comparison and evaluation of approaches aimed at correcting or reducing selectivity bias in growth parameter estimates for fishes. Fisheries Research. 2020;225:105464 10.1016/j.fishres.2019.105464

[59] Pardo SA, Cooper AB, Dulvy NK. Avoiding fishy growth curves. Methods in Ecology and Evolution. 2013;4(4):353–60. 10.1111/2041-210x.12020

[60] Campana SE. How reliable are growth back calculations based on otoliths. Canadian Journal of Fisheries and Aquatic Sciences. 1990;47(11):2219–27. ISI:A1990EG74400016.

[61] Emmons S, Simpfendorfer C, Smart J, D’Alberto B. Age and growth of tiger shark (*Galeocerdo cuvier*) from Western Australia. Marine and Freshwater Research. in press.

[62] Caltabellotta FP, Siders ZA, Cailliet GM, Motta FS, Gadig OBF. Preliminary age and growth of the deep-water goblin shark Mitsukurina owstoni (Jordan, 1898). Marine and Freshwater Research. 2020 10.1071/mf19370

[63] Wilson KL, Honsey AE, Moe B, Venturelli P. Growing the biphasic framework: Techniques and recommendations for fitting emerging growth models. Methods in Ecology and Evolution. 2018;9(4):822–33. 10.1111/2041-210x.12931

[64] Wilson KL, De Gisi J, Cahill CL, Barker OE, Post JR. Life‐history variation along environmental and harvest clines of a northern freshwater fish: Plasticity and adaptation. Journal of Animal Ecology. 2019;88(5):717–33. 10.1111/1365-2656.12965 30784045

[65] Morat F, Wicquart J, Schiettekatte NMD, De Sinéty G, Bienvenu J, Casey JM, et al Individual back-calculated size-at-age based on otoliths from Pacific coral reef fish species. Scientific Data. 2020;7(1). 10.1038/s41597-020-00711-y 33110081 PMC7591892

[66] Matthias BG, Ahrens RNM, Allen MS, Tuten T, Siders ZA, Wilson KL. Understanding the effects of density and environmental variability on the process of fish growth. Fisheries Research. 2018;198:209–19. 10.1016/j.fishres.2017.08.018

