# Supplementary material for: Modernising fish and shark growth curves with Bayesian length-at-age models
Source: PLoS One. 2021 Feb 8;16(2):e0246734. doi: 10.1371/journal.pone.0246734 (PMC7870076; doi:10.1371/journal.pone.0246734)
Supplement: S1 Appendix — (HTML) [file pone.0246734.s001.html]

Appendix 1: Bayesian and frequentist analyses of case studies


# Appendix 1: Bayesian and frequentist analyses of case studies

```
library(tidyverse)
library(BayesGrowth)
library(AquaticLifeHistory)
library(tidybayes)
library(bayesplot)
library(rstan)
library(pander)
library(cowplot)
```

## Silvertip shark

The raw data and the L0 priors based on mean and sd of age zero lengths in Back calculated data

```
head(silvertips)
```

```
##   Age   Length Sex
## 1  18 250.0000   F
## 2  17 239.0000   F
## 3  18 220.0000   F
## 4  16 219.3145   M
## 5  14 218.0000   F
## 6  18 217.0000   F
```

```
silvertips_BC
```

```
##         L0    L0.se
## 1 69.99011 7.094058
```

### Frequentist results

```
Silvertip_nls_results <- Estimate_Growth(silvertips, models = "VB", plots = FALSE)

Silvertips_AIC <- Estimate_Growth(silvertips, n.bootstraps = 0, plots = FALSE)$AIC

pander(Silvertip_nls_results$VonB)
```

|  | Parameter | SE |
| --- | --- | --- |
| **Linf** | 899.4 | 1429 |
| **k** | 0.009321 | 0.01832 |
| **L0** | 100.7 | 6.361 |
| **RSE** | 10.28 | NA |

```
pander(Silvertips_AIC)
```

| Model | AICc | AIC diff | Weight |
| --- | --- | --- | --- |
| VB | 365.8 | 0 | 0.36 |
| Log | 366.1 | 0.3 | 0.31 |
| Gom | 365.9 | 0.16 | 0.33 |

### MCMC results

```
Silvertip_MCMC_results <- Estimate_MCMC_Growth(silvertips,
                                Model = "VB" ,
                                iter = 10000,
                                n.chains = 4,
                                BurnIn = 5000,
                                thin = 1,
                                Linf = 300,
                                Linf.se = 30,
                                L0 = 70,
                                L0.se = 7,
                                sigma.max = 100,
                                verbose = T,
                                n_cores = 3,
                                k.max = 0.3)

silvertip_LooIC <- Compare_Growth_Models(silvertips,
                                iter = 10000,
                                n.chains = 4,
                                BurnIn = 5000,
                                thin = 1,
                                Linf = 300,
                                Linf.se = 30,
                                L0 = 68,
                                L0.se = 5,
                                sigma.max = 100,
                                verbose = T,
                                n_cores = 3,
                                k.max = 0.3)
```

```
pander(Get_MCMC_parameters(Silvertip_MCMC_results))
```

| Parameter | mean | se\_mean | sd | 2.5% | 50% | 97.5% | n\_eff | Rhat |
| --- | --- | --- | --- | --- | --- | --- | --- | --- |
| Linf | 307.1 | 0.23 | 23.87 | 264.4 | 305.9 | 357.7 | 11233 | 1 |
| k | 0.05 | 0 | 0.01 | 0.04 | 0.05 | 0.08 | 10585 | 1 |
| L0 | 82.65 | 0.04 | 4.76 | 72.94 | 82.78 | 91.54 | 14632 | 1 |
| sigma | 11.27 | 0.01 | 1.25 | 9.12 | 11.17 | 14.02 | 18671 | 1 |

```
pander(silvertip_LooIC)
```

Table continues below


| Model | elpd\_diff | se\_diff | elpd\_loo | se\_elpd\_loo | p\_loo | se\_p\_loo |
| --- | --- | --- | --- | --- | --- | --- |
| VB | 0 | 0 | -787.8 | 8.403 | 3.068 | 0.3631 |
| Gompertz | -0.1456 | 2.931 | -788 | 8.245 | 3.652 | 0.4411 |
| Logistic | -3.608 | 3.912 | -791.4 | 8.43 | 4.043 | 0.5406 |

| looic | se\_looic | looic\_Weight |
| --- | --- | --- |
| 1576 | 16.81 | 0.52 |
| 1576 | 16.49 | 0.48 |
| 1583 | 16.86 | 0 |

### Diagnostic plots

Check for chain convergence and that posteriors are not multimodal. Then check that no auto correlation is present

```
mcmc_combo(Silvertip_MCMC_results,pars = c("Linf", "k", "L0", "sigma"))
```

```
mcmc_acf(Silvertip_MCMC_results,pars = c("Linf", "k", "L0", "sigma"))
```

### Growth curves

```
growth_curve <- Calculate_MCMC_growth_curve(Silvertip_MCMC_results, Model = "VB",
                                            max.age = 35, probs = c(.95))

nls_curve <- data.frame(Age = 0:35, AVG = Calc_VBGF_LAA(Silvertip_nls_results$VonB["Linf","Parameter"], 
                                                        Silvertip_nls_results$VonB["k","Parameter"],
                                                        Silvertip_nls_results$VonB["L0","Parameter"],
                                                        0:35))

Silvertip_plot <- ggplot(growth_curve, aes(Age, LAA))+
  ggtitle("Silvertip Shark")+
  geom_line(data = nls_curve,inherit.aes = FALSE,
            aes(Age, AVG, col = "Frequentist model"), size = 1.5)+
  geom_point(data = silvertips, aes(Age, Length), alpha = .3)+
  geom_lineribbon( aes(ymin = .lower, ymax =.upper,fill = "MCMC model", col = "MCMC model"),size = 1.5, alpha = .5) +
  geom_line(aes(Age, LAA, col = "MCMC model"), size = 1.5)+
  labs(y = "Total Length (cm)", x = "Age (yrs)")+
  scale_color_viridis_d(name = "Model",direction = -1, begin = .3, end = .8)+
  scale_fill_viridis_d(begin = .5, end = .6)+
  guides(fill = FALSE)+
  expand_limits(y = 0)+
  scale_y_continuous(expand = c(0,0), breaks = seq(0,300,50))+
  scale_x_continuous(expand = c(0,0), breaks = seq(0,max(growth_curve$Age),5))+
  theme_bw()+
  guides(fill = FALSE, col = FALSE)+
   theme(
        text = element_text(size = 14),
        axis.title = element_blank(),
        plot.title = element_text(hjust = .5),
        legend.background = element_rect(colour = "black"))

Silvertip_plot
```

## Ocean perch

The raw data

```
head(Ocean_perch)
```

```
##   Age Length    Sex
## 1   3    162   Male
## 2   4    179 Female
## 3   5    200 Female
## 4   5    201   Male
## 5   5    178 Female
## 6   6    160
```

### Frequentist results

```
Perch_nls_results <- Estimate_Growth(Ocean_perch, models = "Log", plots = FALSE)

Perch_AIC <- Estimate_Growth(Ocean_perch, n.bootstraps = 0, plots = FALSE)$AIC

pander(Perch_nls_results$Logistic)
```

|  | Parameter | SE |
| --- | --- | --- |
| **Linf** | 304.9 | 4.983 |
| **g** | 0.2713 | 0.0332 |
| **L0** | 84.37 | 13.56 |
| **RSE** | 22.1 | NA |

```
pander(Perch_AIC)
```

| Model | AICc | AIC diff | Weight |
| --- | --- | --- | --- |
| VB | 1392 | 5.32 | 0.05 |
| Log | 1387 | 0 | 0.72 |
| Gom | 1389 | 2.29 | 0.23 |

### MCMC results

```
Perch_MCMC_results <- Estimate_MCMC_Growth(Ocean_perch,
                                Model = "VB" ,
                                iter = 10000,
                                n.chains = 4,
                                BurnIn = 5000,
                                thin = 1,
                                Linf = 470,
                                Linf.se = 5,
                                L0 = 10,
                                L0.se = 0.1,
                                sigma.max = 100,
                                verbose = T,
                                n_cores = 3,
                                k.max = 5)

Perch_LooIC <- Compare_Growth_Models(Ocean_perch,
                                iter = 10000,
                                n.chains = 4,
                                BurnIn = 5000,
                                thin = 1,
                                Linf = 470,
                                Linf.se = 5,
                                L0 = 10,
                                L0.se = 0.1,
                                sigma.max = 100,
                                verbose = T,
                                n_cores = 3,
                                k.max = 5)
```

```
pander(Get_MCMC_parameters(Perch_MCMC_results))
```

| Parameter | mean | se\_mean | sd | 2.5% | 50% | 97.5% | n\_eff | Rhat |
| --- | --- | --- | --- | --- | --- | --- | --- | --- |
| Linf | 462.9 | 0.04 | 5.14 | 452.9 | 462.9 | 473 | 20038 | 1 |
| k | 0.07 | 0 | 0 | 0.06 | 0.07 | 0.07 | 20160 | 1 |
| L0 | 10 | 0 | 0.1 | 9.81 | 10.01 | 10.2 | 34079 | 1 |
| sigma | 34.27 | 0.01 | 2.02 | 30.61 | 34.17 | 38.51 | 28222 | 1 |

```
pander(Perch_LooIC)
```

Table continues below


| Model | elpd\_diff | se\_diff | elpd\_loo | se\_elpd\_loo | p\_loo | se\_p\_loo |
| --- | --- | --- | --- | --- | --- | --- |
| VB | 0 | 0 | -758.1 | 8.708 | 1.923 | 0.3292 |
| Gompertz | -81.58 | 3.854 | -839.7 | 7.726 | 1.658 | 0.1835 |
| Logistic | -141.2 | 5.131 | -899.3 | 6.002 | 1.384 | 0.07861 |

| looic | se\_looic | looic\_Weight |
| --- | --- | --- |
| 1516 | 17.42 | 1 |
| 1679 | 15.45 | 0 |
| 1799 | 12 | 0 |

### Diagnostic plots

Check for chain convergence and that posteriors are not multimodal. Then check that no auto correlation is present

```
mcmc_combo(Perch_MCMC_results,pars = c("Linf", "k", "L0", "sigma"))
```

```
mcmc_acf(Perch_MCMC_results,pars = c("Linf", "k", "L0", "sigma"))
```

### Growth curves

```
growth_curve <- Calculate_MCMC_growth_curve(Perch_MCMC_results, Model = "VB",
                                            max.age = 45, probs = c(.95))

nls_curve <- data.frame(Age = 0:45, AVG = Calc_Logistic_LAA(Perch_nls_results$Logistic["Linf","Parameter"], 
                                                            Perch_nls_results$Logistic["g","Parameter"],
                                                            Perch_nls_results$Logistic["L0","Parameter"],
                                                            0:45))

Perch_plot <- ggplot(growth_curve, aes(Age, LAA))+
   ggtitle("Reef Ocean Perch")+
  geom_line(data = nls_curve,inherit.aes = FALSE,
            aes(Age, AVG, col = "Frequentist model"), size = 1.5)+
  geom_point(data = Ocean_perch, aes(Age, Length), alpha = .3)+
  geom_lineribbon( aes(ymin = .lower, ymax =.upper,fill = "MCMC model", col = "MCMC model"),size = 1.5, alpha = .5) +
  geom_line(aes(Age, LAA, col = "MCMC model"), size = 1.5)+
  labs(y = "Total Length (cm)", x = "Age (yrs)")+
  scale_color_viridis_d(name = "Model",direction = -1, begin = .3, end = .8)+
  scale_fill_viridis_d(begin = .5, end = .6)+
  guides(fill = FALSE)+
  scale_y_continuous(expand = c(0,0), labels = seq(0,40,10))+
  scale_x_continuous(expand = c(0,0), breaks = seq(0,42,5))+
  guides(fill = FALSE, col = FALSE)+
  theme_bw()+
    theme(
        text = element_text(size = 14),
        plot.title = element_text(hjust = .5),
        legend.background = element_rect(colour = "black"))

Perch_plot
```

## Silky shark

```
head(silky)
```

```
##     tag Age Length Sex
## 1 10001  12    178   F
## 2 10002  13    178   M
## 3 10003  16    217   M
## 4 10004  10    165   F
## 5 10005   8    139   M
## 6 10006  10    170   M
```

### Frequentist results

```
Silky_nls_results <- Estimate_Growth(silky, models = "Log", plots = FALSE)

Silkys_AIC <- Estimate_Growth(silky, n.bootstraps = 0, plots = FALSE)$AIC

pander(Silky_nls_results$Logistic)
```

|  | Parameter | SE |
| --- | --- | --- |
| **Linf** | 268.3 | 6.058 |
| **g** | 0.1361 | 0.006093 |
| **L0** | 82.59 | 1.714 |
| **RSE** | 14.64 | NA |

```
pander(Silkys_AIC)
```

| Model | AICc | AIC diff | Weight |
| --- | --- | --- | --- |
| VB | 4348 | 26.94 | 0 |
| Log | 4321 | 0 | 1 |
| Gom | 4332 | 11.09 | 0 |

### MCMC results

```
Silky_MCMC_results <- Estimate_MCMC_Growth(silky,
                                Model = "Log" ,
                                iter = 10000,
                                n.chains = 4,
                                BurnIn = 5000,
                                thin = 1,
                                Linf = 280,
                                Linf.se = 15,
                                L0 = 77,
                                L0.se = 5,
                                sigma.max = 100,
                                verbose = T,
                                n_cores = 3,
                                k.max = 5)


Silky_LooIC <- Compare_Growth_Models(silky,
                                iter = 10000,
                                n.chains = 4,
                                BurnIn = 5000,
                                thin = 1,
                                Linf = 280,
                                Linf.se = 15,
                                L0 = 77,
                                L0.se = 5,
                                sigma.max = 100,
                                verbose = T,
                                n_cores = 3,
                                k.max = 5)
```

```
pander(Get_MCMC_parameters(Silky_MCMC_results))
```

| Parameter | mean | se\_mean | sd | 2.5% | 50% | 97.5% | n\_eff | Rhat |
| --- | --- | --- | --- | --- | --- | --- | --- | --- |
| Linf | 269.1 | 0.05 | 5.41 | 259.1 | 268.9 | 280.3 | 13631 | 1 |
| k | 0.14 | 0 | 0.01 | 0.13 | 0.14 | 0.15 | 12105 | 1 |
| L0 | 82.32 | 0.01 | 1.57 | 79.23 | 82.31 | 85.42 | 14156 | 1 |
| sigma | 14.67 | 0 | 0.45 | 13.83 | 14.66 | 15.6 | 17793 | 1 |

```
pander(Silky_LooIC)
```

Table continues below


| Model | elpd\_diff | se\_diff | elpd\_loo | se\_elpd\_loo | p\_loo | se\_p\_loo |
| --- | --- | --- | --- | --- | --- | --- |
| Logistic | 0 | 0 | -2161 | 27.56 | 5.443 | 1.591 |
| Gompertz | -5.478 | 1.691 | -2167 | 27.34 | 5.24 | 1.576 |
| VB | -17.24 | 4.247 | -2178 | 26.56 | 4.657 | 1.52 |

| looic | se\_looic | looic\_Weight |
| --- | --- | --- |
| 4322 | 55.11 | 1 |
| 4333 | 54.67 | 0 |
| 4357 | 53.13 | 0 |

### Diagnostic plots

Check for chain convergence and that posteriors are not multimodal. Then check that no auto correlation is present

```
mcmc_combo(Silky_MCMC_results,pars = c("Linf", "k", "L0", "sigma"))
```

```
mcmc_acf(Silky_MCMC_results,pars = c("Linf", "k", "L0", "sigma"))
```

### Growth curves

```
growth_curve <- Calculate_MCMC_growth_curve(Silky_MCMC_results, Model = "Log",
                                            max.age = 27, probs = c(.95))

nls_curve <- data.frame(Age = 0:27, AVG = Calc_Logistic_LAA(Silky_nls_results$Logistic["Linf","Parameter"], 
                                                            Silky_nls_results$Logistic["g","Parameter"],
                                                            Silky_nls_results$Logistic["L0","Parameter"],
                                                            0:27))

Silky_plot <- ggplot(growth_curve, aes(Age, LAA))+
    ggtitle("Silky Shark")+
  geom_line(data = nls_curve,inherit.aes = FALSE,
            aes(Age, AVG, col = "Frequentist model"), size = 1.5)+
  geom_point(data = silky, aes(Age, Length), alpha = .3)+
  geom_lineribbon( aes(ymin = .lower, ymax =.upper,fill = "MCMC model", col = "MCMC model"),size = 1.5, alpha = .5) +
  geom_line(aes(Age, LAA, col = "MCMC model"), size = 1.5)+
  labs(y = "Total Length (cm)", x = "Age (yrs)")+
  scale_color_viridis_d(name = "Model",direction = -1, begin = .3, end = .8)+
  scale_fill_viridis_d(begin = .5, end = .6)+
  guides(fill = FALSE)+
  scale_y_continuous(expand = c(0,0))+
  scale_x_continuous(expand = c(0,0), breaks = seq(0,max(growth_curve$Age),5))+
  theme_bw()+
  guides(fill = FALSE, col = FALSE)+
   theme(
        text = element_text(size = 14),
        axis.title.y = element_blank(),
        plot.title = element_text(hjust = .5),
        legend.background = element_rect(colour = "black"))

Silky_plot
```

## Blue Mackerel

```
head(Blue_mack)
```

```
##   Length      Age
## 1    310 8.652071
## 2    271 3.652071
## 3    275 3.652071
## 4    302 3.652071
## 5    281 3.652071
## 6    248 3.652071
```

### Frequentist results

**Free L0**

```
Blue_mack_nls_results <- Estimate_Growth(Blue_mack, models = "VB", plots = FALSE)

Blue_mack_AIC <- Estimate_Growth(Blue_mack, n.bootstraps = 0, plots = FALSE)$AIC

pander(Blue_mack_nls_results$VonB)
```

|  | Parameter | SE |
| --- | --- | --- |
| **Linf** | 378.4 | 23.73 |
| **k** | 0.1479 | 0.03627 |
| **L0** | 209.1 | 5.887 |
| **RSE** | 19.42 | NA |

```
pander(Blue_mack_AIC)
```

| Model | AICc | AIC diff | Weight |
| --- | --- | --- | --- |
| VB | 6925 | 0 | 0.69 |
| Log | 6929 | 4.3 | 0.08 |
| Gom | 6927 | 2.17 | 0.23 |

**Fixed L0 at zero**

Only VB model converges

```
fixed_Blue_mack_nls_results<- Estimate_Growth(Blue_mack, Birth.Len = 0, models = "VB", plots = FALSE)

pander(fixed_Blue_mack_nls_results$VonB)
```

|  | Parameter | SE |
| --- | --- | --- |
| **Linf** | 292.7 | 1.314 |
| **k** | 0.9878 | 0.03067 |
| **RSE** | 21.75 | NA |

```
Blue_mack_nls_results <- Estimate_Growth(Blue_mack, models = "VB", plots = FALSE)

Blue_mack_AIC <- Estimate_Growth(Blue_mack, n.bootstraps = 0, plots = FALSE)$AIC
Blue_mack_MCMC_results <-  Estimate_MCMC_Growth(Blue_mack,
                                Model = "VB" ,
                                iter = 10000,
                                n.chains = 4,
                                BurnIn = 5000,
                                thin = 10,
                                Linf = 440,
                                Linf.se = 5,
                                L0 = 0,
                                L0.se = 0.001,
                                sigma.max = 100,
                                verbose = T,
                                n_cores = 3,
                                k.max = 1)

Blue_mack_LooIC <- Compare_Growth_Models(Blue_mack,
                                iter = 10000,
                                n.chains = 4,
                                BurnIn = 5000,
                                thin = 1,
                                Linf = 440,
                                Linf.se = 5,
                                L0 = 0,
                                L0.se = 0.001,
                                sigma.max = 100,
                                verbose = T,
                                n_cores = 3,
                                k.max = 1)
```

### Diagnostic plots

Check for chain convergence and that posteriors are not multimodal. Then check that no auto correlation is present

```
mcmc_combo(Blue_mack_MCMC_results,pars = c("Linf", "k", "L0", "sigma"))
```

```
mcmc_acf(Blue_mack_MCMC_results,pars = c("Linf", "k", "L0", "sigma"))
```

### MCMC results

```
pander(Get_MCMC_parameters(Blue_mack_MCMC_results))
```

| Parameter | mean | se\_mean | sd | 2.5% | 50% | 97.5% | n\_eff | Rhat |
| --- | --- | --- | --- | --- | --- | --- | --- | --- |
| Linf | 318 | 0.07 | 4.15 | 310.9 | 317.7 | 327 | 3641 | 1 |
| k | 0.66 | 0 | 0.03 | 0.59 | 0.66 | 0.73 | 3466 | 1 |
| L0 | 0 | 0 | 0 | 0 | 0 | 0 | 3010 | 1 |
| sigma | 24.31 | 0.02 | 0.89 | 22.72 | 24.27 | 26.18 | 3493 | 1 |

```
pander(Blue_mack_LooIC)
```

Table continues below


| Model | elpd\_diff | se\_diff | elpd\_loo | se\_elpd\_loo | p\_loo | se\_p\_loo |
| --- | --- | --- | --- | --- | --- | --- |
| VB | 0 | 0 | -3641 | 33.08 | 8.865 | 1.476 |
| Gompertz | -3669 | 40.17 | -7310 | 16.8 | 0.2958 | 0.02751 |
| Logistic | -3832 | 40.65 | -7473 | 20.21 | 0.001686 | 3.736e-05 |

| looic | se\_looic | looic\_Weight |
| --- | --- | --- |
| 7282 | 66.16 | 1 |
| 14620 | 33.6 | 0 |
| 14946 | 40.42 | 0 |

### Growth curves

```
growth_curve <- Calculate_MCMC_growth_curve(Blue_mack_MCMC_results, Model = "VB",
                                            max.age = 12, probs = c(.95))

nls_curve <- data.frame(Age = 0:12, AVG = Calc_VBGF_LAA(Blue_mack_nls_results$VonB["Linf","Parameter"], 
                                                        Blue_mack_nls_results$VonB["k","Parameter"],
                                                        Blue_mack_nls_results$VonB["L0","Parameter"],
                                                        0:12))


fixed_nls_curve <- data.frame(Age = seq(0,12,0.1), AVG = Calc_VBGF_LAA(fixed_Blue_mack_nls_results$VonB["Linf","Parameter"], 
                                                                       fixed_Blue_mack_nls_results$VonB["k","Parameter"],
                                                                       0,
                                                                       seq(0,12,0.1)))

Blue_mack_plot <- ggplot(growth_curve, aes(Age, LAA))+
   ggtitle("Blue Mackerel")+
  geom_line(data = fixed_nls_curve,inherit.aes = FALSE,
            aes(Age, AVG, col = "Frequentist model - Fixed L0"), size = 1.5)+
  geom_line(data = nls_curve,inherit.aes = FALSE,
            aes(Age, AVG, col = "Frequentist model - Free L0"), size = 1.5)+
  geom_point(data = Blue_mack, aes(Age, Length), alpha = .3)+
  geom_lineribbon( aes(ymin = .lower, ymax =.upper,fill = "Bayesian model", col = "Bayesian model"),
                   size = 1.5, alpha = .5) +
  geom_line(aes(Age, LAA, col = "Bayesian model"), size = 1.5)+
  labs(y = "Total Length (cm)", x = "Age (yrs)")+
  scale_colour_manual(name = "Model", values = c("#35608DFF","#1E9C89FF","#7AD151FF" ))+
  scale_fill_viridis_d(begin = .5, end = .6)+
  guides(fill = FALSE)+
  scale_y_continuous(expand = c(0,0), labels = seq(0,40,10))+
  scale_x_continuous(expand = c(0,0), breaks = seq(0,max(growth_curve$Age),1))+
  theme_bw()+
  theme(legend.position = c(0.6,0.2),
        text = element_text(size = 14),
        axis.title.x = element_blank(),
        plot.title = element_text(hjust = .5),
        legend.background = element_rect(colour = "black"))

Blue_mack_plot
```
