# Supplementary material for: Modernising fish and shark growth curves with Bayesian length-at-age models
Source: PLoS One. 2021 Feb 8;16(2):e0246734. doi: 10.1371/journal.pone.0246734 (PMC7870076; doi:10.1371/journal.pone.0246734)
Supplement: S2 Appendix — (HTML) [file pone.0246734.s002.html]

Appendix 2: Bayesian vs frequentist growth model comparisons using simulated gear selectivities


# Appendix 2: Bayesian vs frequentist growth model comparisons using simulated gear selectivities

## Load libaries

```
library(tidyverse)
library(truncnorm)
library(AquaticLifeHistory)
library(BayesGrowth)
library(tidybayes)
library(bayesplot)
library(tmvtnorm)
library(cowplot)
library(pander)

theme_set(theme_bw())
```

## Selectivity functions

These are the functions used in this analysis to simulate the length-at-age data in one of three scenarios:

- Dome shaped selectivity
- Selectivity that decreases with age (referred to as young\_selectivity)
- Selectivity that increases with age (referred to asold\_selectivity)

```
dome_selectivity <- function(Age, 
                             mean_sel, 
                             sd_sel, 
                             M =.2, 
                             n = 100,
                             Linf, 
                             k, 
                             L0, 
                             sigma=Linf/10) {
  addTaskCallback(function(...) {set.seed(123);TRUE})
  selectivity <- dnorm(Age,mean_sel,sd_sel)/max(dnorm(Age,mean_sel,sd_sel))
  survival <- rep(NA, length(Age)) 
  survival[1] <- 1
  for (i in 2:(max(Age)+1)) {
    survival[i] <- survival[i - 1] * exp(-M)
  } 
  
  sims <- as.vector(rmultinom(1, prob = survival * selectivity, size = 10000))
  
  age_structure <- sims/max(sims)
  
  Observed_lengths <-  Linf-(Linf-L0)*(exp(-k*Age))
  
  
  Sampled_lengths <- as.data.frame(cbind(Observed_lengths, sims))  
  Selected_lengths <- rep(Sampled_lengths[, 1], Sampled_lengths[, 2])  
  Age_col <- rep(Age, Sampled_lengths[, 2]) 
  
  
  len_col <- sapply(Selected_lengths, function(x) rtruncnorm(1,a=-0.0001, mean = x, sd = x^sigma))
  
  
  Results <- as.data.frame(cbind(Age_col, len_col))  
  colnames(Results) <- c("Age", "Lt")  
  Results <-Results[sample(nrow(Results), size = n, replace = T), ] 
  
  return(list(LAA = Results, survival=survival, selectivity=selectivity, `Relative sampled ages` = age_structure))
}
```

```
young_selectivity <- function(Age, 
                              mean_sel, 
                              slope_sel, 
                              M =.2, 
                              n = 100,
                              Linf, 
                              k, 
                              L0, 
                              sigma=Linf/10) {
  
  addTaskCallback(function(...) {set.seed(2020);TRUE})
  
  
  selectivity <- 1/(1 + exp(abs(slope_sel) * (Age - mean_sel)))
  survival <- rep(NA, length(Age)) 
  survival[1] <- 1
  for (i in 2:(max(Age)+1)) {
    survival[i] <- survival[i - 1] * exp(-M)
  } 
  
  sims <- as.vector(rmultinom(1, prob = survival * selectivity, size = 10000))
  
  age_structure <- sims/max(sims)
  
  
  Observed_lengths <-  Linf-(Linf-L0)*(exp(-k*Age))
  
  
  Sampled_lengths <- as.data.frame(cbind(Observed_lengths, sims))  
  Selected_lengths <- rep(Sampled_lengths[, 1], Sampled_lengths[, 2])  
  Age_col <- rep(0:max(Age), Sampled_lengths[, 2]) 
  
  
  len_col <- sapply(Selected_lengths, function(x) rtruncnorm(1,a=-0.0001, mean = x, sd = x^sigma))
  
  
  Results <- as.data.frame(cbind(Age_col, len_col))  
  colnames(Results) <- c("Age", "Lt")  
  Results <-Results[sample(nrow(Results), size = n, replace = F), ] 
  
  return(list(LAA = Results, survival=survival, selectivity=selectivity, `Relative sampled ages` = age_structure))
}
```

```
old_selectivity <- function(Age, 
                            mean_sel, 
                            slope_sel, 
                            M =.2, 
                            n = 100,
                            Linf, 
                            k, 
                            L0, 
                            sigma=Linf/10) {
  addTaskCallback(function(...) {set.seed(123);TRUE})
  
  selectivity <- 1/(1 + exp(-abs(slope_sel) * (Age - mean_sel)))
  survival <- rep(NA, length(Age))
  survival[1] <- 1
  for (i in 2:(max(Age)+1)) {
    survival[i] <- survival[i - 1] * exp(-M)
  } 
  
  sims <- as.vector(rmultinom(1, prob = survival * selectivity, size = 10000))
  
  age_structure <- (survival * selectivity)/max((survival * selectivity))
  
  Observed_lengths <-  Linf-(Linf-L0)*(exp(-k*Age))
  
  
  Sampled_lengths <- as.data.frame(cbind(Observed_lengths, sims))  
  Selected_lengths <- rep(Sampled_lengths[, 1], Sampled_lengths[, 2])  
  Age_col <- rep(Age, Sampled_lengths[, 2]) 
  
  
  len_col <- sapply(Selected_lengths, function(x) rtruncnorm(1,a=-0.0001, mean = x, sd = x^sigma))
  
  
  Results <- as.data.frame(cbind(Age_col, len_col))  
  colnames(Results) <- c("Age", "Lt")  
  Results <-Results[sample(nrow(Results), size = n, replace = T), ] 
  
  return(list(LAA = Results, survival=survival, selectivity=selectivity, `Relative sampled ages` = age_structure))
}
```

# Scenarios

These are the life history parameters used in all scenarios. Sigma is multiplicative such that the residuals increase proportionally with length. A mortality rate of 0.2 is applied in all scenarios. The `real_curve` is the target growth curve that each model is attempting to estimate.

```
# Global parameters for all scenarios

Linf_obs <- 250
k_obs <- 0.2
L0_obs <- 0
sigma_obs <- 0.5
max_age <- 20

real_curve <- data.frame(Age = 0:max_age, AVG = Calc_VBGF_LAA(Linf_obs, 
                                                              k_obs,
                                                              L0_obs,
                                                              0:max_age))
```

## Missing young individuals

This scenario represents a logistic selectivity function where fishing gear catches older individuals but not younger ones. Therefore, L0 is often poorly estimated. The logistic function was set to have an S50 of 12 years with a slope of 0.75.

### Simulate data and plot conditions

```
old_sel_results_50 <- old_selectivity(M = .2, Age = 0:max_age, slope_sel = 0.75,
                                      mean_sel = 12, n = 50, Linf = Linf_obs, k = k_obs , L0 = L0_obs, sigma = sigma_obs)

old_sel_LAA_50 <- old_sel_results_50[[1]]

old_sel_results_100 <- old_selectivity(M = .2, Age = 0:max_age, slope_sel = 0.75,
                                       mean_sel = 12, n = 100, Linf = Linf_obs, k = k_obs , L0 = L0_obs, sigma = sigma_obs)

old_sel_LAA_100 <- old_sel_results_100[[1]]


old_sel_results_150 <- old_selectivity(M = .2, Age = 0:max_age, slope_sel = 0.75,
                                       mean_sel = 12, n = 150, Linf = Linf_obs, k = k_obs , L0 = L0_obs, sigma = sigma_obs)


old_sel_LAA_150 <- old_sel_results_150[[1]]


old_sel_conditions <-  data.frame(Age = 0:20,
                                  survival = old_sel_results_50[["survival"]] ,
                                  selectivity=  old_sel_results_50[["selectivity"]],
                                  `Relative sampled ages` = old_sel_results_50[["Relative sampled ages"]]) %>%
  set_names(c("Age", "Survival", "Selectivity", "Relative Sampled Ages")) %>%
  pivot_longer(-Age, names_to = "Type",values_to = "Values" )
old_sel_conditions$Type <- factor(old_sel_conditions$Type,levels = c("Survival","Selectivity","Relative Sampled Ages"))

old_sel_cond_plot <- ggplot(old_sel_conditions, aes(x = Age, y = Values, fill = Type, col = Type))+
  ggtitle(" ")+
  geom_area(size = 1, position = position_dodge(), alpha = .5)+
  scale_fill_viridis_d(direction = -1)+
  scale_colour_viridis_d(direction = -1)+
  labs(y = "Proportion", x = "Age (years)")+
  scale_x_continuous(expand = c(0,0))+
  scale_y_continuous(expand = c(0,0))+
  theme_bw()+
  theme(legend.position ="none")

old_sel_cond_plot
```

### Fit models to simulation where n = 50

```
Bayes_results_old_50 <- Estimate_MCMC_Growth(data = old_sel_LAA_50,
                                             Model = "VB",
                                             Linf = 275,
                                             k.max = 0.5,
                                             L0 = 0,
                                             L0.se = 0.01,
                                             Linf.se = 27.5,
                                             sigma.max = 50,
                                             BurnIn = 5000,
                                             n_cores = 3,
                                             iter = 10000)
```

```
nls_results_old_50 <- Estimate_Growth(data = old_sel_LAA_50, models = "VB",n.bootstraps = 1000, plots = F,Max.Age = max_age)

Bayes_curve_old_50 <- Calculate_MCMC_growth_curve(Bayes_results_old_50,Model = "VB", max.age = max(nls_results_old_50$Estimates$Age), probs = 0.95)


old_sel_50_plot <- ggplot() +
  geom_point( data = old_sel_LAA_50, aes(x = Age, y = Lt), alpha = 0.3)+
  labs(y = "Length (cm)", x = "Age (years)")+
  geom_ribbon(data = nls_results_old_50$Estimates, aes(x = Age, ymin = low, ymax = upp, fill = "Frequentist"),alpha = .5 )+
  geom_line(data = nls_results_old_50$Estimates, aes(x = Age, y = AVG,col = "Frequentist"),alpha = .5, size = 1.2)+
  geom_lineribbon(data = Bayes_curve_old_50, aes(x = Age, y = LAA, ymin = .lower, ymax = .upper,fill = "Bayesian",col = "Bayesian"),
                  alpha = .5)+
  geom_line(data = real_curve, aes(x = Age, y = AVG),col = "blue", linetype = "dashed", size = 1.2)+
  scale_x_continuous(expand = c(0,0))+
  scale_y_continuous(expand = c(0,0))+
  scale_fill_viridis_d(end = 0.5, name = "Model")+
  scale_colour_viridis_d(end = 0.5, guide = FALSE)+
  theme(legend.position ="none")

pander(Get_MCMC_parameters(Bayes_results_old_50),caption = "MCMC - selectivity increases with age")
```

MCMC - selectivity increases with age


| Parameter | mean | se\_mean | sd | 2.5% | 50% | 97.5% | n\_eff | Rhat |
| --- | --- | --- | --- | --- | --- | --- | --- | --- |
| Linf | 248.7 | 0.07 | 6.13 | 237.8 | 248.3 | 262 | 7350 | 1 |
| k | 0.21 | 0 | 0.03 | 0.17 | 0.21 | 0.27 | 7395 | 1 |
| L0 | 0.01 | 0 | 0.01 | 0 | 0.01 | 0.02 | 11613 | 1 |
| sigma | 15.68 | 0.02 | 1.66 | 12.83 | 15.54 | 19.34 | 11121 | 1 |

### Fit models to simulation where n = 100

```
Bayes_results_old_100 <- Estimate_MCMC_Growth(data = old_sel_LAA_100,
                                              Model = "VB",
                                              Linf = 275,
                                              k.max = 0.5,
                                              L0 = 0,
                                              L0.se = 0.01,
                                              Linf.se = 27.5,
                                              sigma.max = 50,
                                              BurnIn = 5000,
                                              n_cores = 3,
                                              thin = 100,
                                              iter = 10000)
```

```
nls_results_old_100 <- Estimate_Growth(data = old_sel_LAA_100, models = "VB",n.bootstraps = 1000, plots = F,Max.Age = max_age)


Bayes_curve_old_100 <- Calculate_MCMC_growth_curve(Bayes_results_old_100,Model = "VB", max.age = max(nls_results_old_100$Estimates$Age), probs = 0.95)


old_sel_100_plot <- ggplot() +
  geom_point( data = old_sel_LAA_100, aes(x = Age, y = Lt), alpha = 0.3)+
  labs(y = "Length (cm)", x = "Age (years)")+
  geom_ribbon(data = nls_results_old_100$Estimates, aes(x = Age, ymin = low, ymax = upp, fill = "Frequentist"),alpha = .5 )+
  geom_line(data = nls_results_old_100$Estimates, aes(x = Age, y = AVG,col = "Frequentist"),alpha = .5, size = 1.2)+
  geom_lineribbon(data = Bayes_curve_old_100, aes(x = Age, y = LAA, ymin = .lower, ymax = .upper,fill = "Bayesian",col = "Bayesian"),
                  alpha = .5)+
  geom_line(data = real_curve, aes(x = Age, y = AVG),col = "blue", linetype = "dashed", size = 1.2)+
  scale_x_continuous(expand = c(0,0))+
  scale_y_continuous(expand = c(0,0))+
  scale_fill_viridis_d(end = 0.5, name = "Model")+
  scale_colour_viridis_d(end = 0.5, guide = FALSE)+
  theme(legend.position ="none")

pander(Get_MCMC_parameters(Bayes_results_old_100),caption = "MCMC - selectivity increases with age")
```

MCMC - selectivity increases with age


| Parameter | mean | se\_mean | sd | 2.5% | 50% | 97.5% | n\_eff | Rhat |
| --- | --- | --- | --- | --- | --- | --- | --- | --- |
| Linf | 252.6 | 0.05 | 4.26 | 244.9 | 252.4 | 261.6 | 8652 | 1 |
| k | 0.2 | 0 | 0.02 | 0.17 | 0.19 | 0.23 | 8774 | 1 |
| L0 | 0.01 | 0 | 0.01 | 0 | 0.01 | 0.02 | 11973 | 1 |
| sigma | 15.15 | 0.01 | 1.08 | 13.23 | 15.08 | 17.44 | 13124 | 1 |

### Fit models to simulation where n = 150

```
Bayes_results_old_150 <- Estimate_MCMC_Growth(data = old_sel_LAA_150,
                                              Model = "VB",
                                              Linf = 275,
                                              k.max = 0.5,
                                              L0 = 0,
                                              L0.se = 0.01,
                                              Linf.se = 27.5,
                                              sigma.max = 50,
                                              BurnIn = 5000,
                                              n_cores = 3,
                                              iter = 10000)
```

```
nls_results_old_150 <- Estimate_Growth(data = old_sel_LAA_150, models = "VB",n.bootstraps = 1000, plots = F,Max.Age = max_age)

Bayes_curve_old_150 <- Calculate_MCMC_growth_curve(Bayes_results_old_150,Model = "VB", max.age = max(nls_results_old_150$Estimates$Age), probs = 0.95)


old_sel_150_plot <- ggplot() +
  geom_point( data = old_sel_LAA_150, aes(x = Age, y = Lt), alpha = 0.3)+
  labs(y = "Length (cm)", x = "Age (years)")+
  geom_ribbon(data = nls_results_old_150$Estimates, aes(x = Age, ymin = low, ymax = upp, fill = "Frequentist"),alpha = .5 )+
  geom_line(data = nls_results_old_150$Estimates, aes(x = Age, y = AVG,col = "Frequentist"),alpha = .5, size = 1.2)+
  geom_lineribbon(data = Bayes_curve_old_150, aes(x = Age, y = LAA, ymin = .lower, ymax = .upper,fill = "Bayesian",col = "Bayesian"),
                  alpha = .5)+
  geom_line(data = real_curve, aes(x = Age, y = AVG),col = "blue", linetype = "dashed", size = 1.2)+
  scale_x_continuous(expand = c(0,0))+
  scale_y_continuous(expand = c(0,0))+
  scale_fill_viridis_d(end = 0.5, name = "Model")+
  scale_colour_viridis_d(end = 0.5, guide = FALSE)+
  theme(legend.position ="none")

pander(Get_MCMC_parameters(Bayes_results_old_150),caption = "MCMC - selectivity increases with age")
```

MCMC - selectivity increases with age


| Parameter | mean | se\_mean | sd | 2.5% | 50% | 97.5% | n\_eff | Rhat |
| --- | --- | --- | --- | --- | --- | --- | --- | --- |
| Linf | 250.6 | 0.04 | 3.5 | 244.2 | 250.4 | 257.9 | 8060 | 1 |
| k | 0.2 | 0 | 0.01 | 0.17 | 0.2 | 0.23 | 8094 | 1 |
| L0 | 0.01 | 0 | 0.01 | 0 | 0.01 | 0.02 | 12160 | 1 |
| sigma | 16.05 | 0.01 | 0.93 | 14.35 | 16.01 | 18.01 | 11530 | 1 |

### Final old sel plots

```
Old_sel_plots <- plot_grid(old_sel_cond_plot,old_sel_50_plot, old_sel_100_plot, old_sel_150_plot, align = "h", nrow = 1,labels = c("Selectivity increases with age", "Sample size = 50", "Sample size = 100", "Sample size = 150"),label_size = 10,label_x = c(-.2,0,0,0))
```

## Dome shaped selectvity

This scenario represents a dome shaped selectivity where older and younger individuals are under represented. A normally distributed selectivity curve was used with a mean age of 10 years and an SD of 2 years. The sample size drawn was 100 individuals.

### Simulate data and plot conditions

```
dome_sel_results_50 <- dome_selectivity(M = .2, Age = 0:max_age, sd_sel = 2,
                                        mean_sel = 10, n = 50, Linf = Linf_obs, k = k_obs , L0 = L0_obs, sigma = sigma_obs)

dome_sel_LAA_50 <- dome_sel_results_50[[1]]


dome_sel_results_100 <- dome_selectivity(M = .2, Age = 0:max_age, sd_sel = 2,
                                         mean_sel = 10, n = 100, Linf = Linf_obs, k = k_obs , L0 = L0_obs, sigma = sigma_obs)

dome_sel_LAA_100 <- dome_sel_results_100[[1]]


dome_sel_results_150 <- dome_selectivity(M = .2, Age = 0:max_age, sd_sel = 2,
                                         mean_sel = 10, n = 150, Linf = Linf_obs, k = k_obs , L0 = L0_obs, sigma = sigma_obs)

dome_sel_LAA_150 <- dome_sel_results_150[[1]]


dome_sel_conditions <-  data.frame(Age = 0:20,
                                   survival = dome_sel_results_50[["survival"]] ,
                                   selectivity=  dome_sel_results_50[["selectivity"]],
                                   `Relative sampled ages` = dome_sel_results_50[["Relative sampled ages"]]) %>%
  set_names(c("Age", "Survival", "Selectivity", "Relative Sampled Ages")) %>%
  pivot_longer(-Age, names_to = "Type",values_to = "Values" )
dome_sel_conditions$Type <- factor(dome_sel_conditions$Type,levels = c("Survival","Selectivity","Relative Sampled Ages"))

dome_sel_cond_plot <- ggplot(dome_sel_conditions, aes(x = Age, y = Values, fill = Type, col = Type))+
  ggtitle(" ")+
  geom_area(size = 1, position = position_dodge(), alpha = .5)+
  scale_fill_viridis_d(direction = -1)+
  scale_colour_viridis_d(direction = -1)+
  labs(y = "Proportion", x = "Age (years)")+
  scale_x_continuous(expand = c(0,0))+
  scale_y_continuous(expand = c(0,0))+
  theme_bw()+
  theme(legend.position ="none")
```

### Fit models to simulation where n = 50

```
Bayes_results_dome_50 <- Estimate_MCMC_Growth(data = dome_sel_LAA_50,
                                              Model = "VB",
                                              Linf = 275,
                                              k.max = 0.5,
                                              L0 = 0,
                                              L0.se = 0.01,
                                              Linf.se = 27.5,
                                              sigma.max = 50,
                                              BurnIn = 5000,
                                              n_cores = 3,
                                              iter = 10000)
```

```
nls_results_dome_50 <- Estimate_Growth(data = dome_sel_LAA_50, models = "VB",n.bootstraps = 1000, plots = F,Max.Age = max_age)

Bayes_curve_dome_50 <- Calculate_MCMC_growth_curve(Bayes_results_dome_50,Model = "VB", max.age = max(nls_results_dome_50$Estimates$Age), probs = 0.95)


dome_sel_50_plot <- ggplot() +
  geom_point( data = dome_sel_LAA_50, aes(x = Age, y = Lt), alpha = 0.3)+
  labs(y = "Length (cm)", x = "Age (years)")+
  geom_ribbon(data = nls_results_dome_50$Estimates, aes(x = Age, ymin = low, ymax = upp, fill = "Frequentist"),alpha = .5 )+
  geom_line(data = nls_results_dome_50$Estimates, aes(x = Age, y = AVG,col = "Frequentist"),alpha = .5, size = 1.2)+
  geom_lineribbon(data = Bayes_curve_dome_50, aes(x = Age, y = LAA, ymin = .lower, ymax = .upper,fill = "Bayesian",col = "Bayesian"),
                  alpha = .5)+
  geom_line(data = real_curve, aes(x = Age, y = AVG),col = "blue", linetype = "dashed", size = 1.2)+
  scale_x_continuous(expand = c(0,0))+
  scale_y_continuous(expand = c(0,0))+
  scale_fill_viridis_d(end = 0.5, name = "Model")+
  scale_colour_viridis_d(end = 0.5, guide = FALSE)+
  theme(legend.position ="none")

pander(Get_MCMC_parameters(Bayes_results_dome_50),caption = "MCMC - selectivity increases with age")
```

MCMC - selectivity increases with age


| Parameter | mean | se\_mean | sd | 2.5% | 50% | 97.5% | n\_eff | Rhat |
| --- | --- | --- | --- | --- | --- | --- | --- | --- |
| Linf | 249.6 | 0.11 | 9.34 | 233.1 | 249 | 269.5 | 7330 | 1 |
| k | 0.19 | 0 | 0.02 | 0.16 | 0.19 | 0.24 | 7217 | 1 |
| L0 | 0.01 | 0 | 0.01 | 0 | 0.01 | 0.02 | 10269 | 1 |
| sigma | 13.34 | 0.01 | 1.38 | 10.96 | 13.22 | 16.36 | 10816 | 1 |

### Fit models to simulation where n = 100

```
Bayes_results_dome_100 <- Estimate_MCMC_Growth(data = dome_sel_LAA_100,
                                               Model = "VB",
                                               Linf = 275,
                                               k.max = 0.5,
                                               L0 = 0,
                                               L0.se = 0.01,
                                               Linf.se = 27.5,
                                               sigma.max = 50,
                                               BurnIn = 5000,
                                               n_cores = 3,
                                               thin = 100,
                                               iter = 10000)
```

```
nls_results_dome_100 <- Estimate_Growth(data = dome_sel_LAA_100, models = "VB",n.bootstraps = 1000, plots = F,Max.Age = max_age)

Bayes_curve_dome_100 <- Calculate_MCMC_growth_curve(Bayes_results_dome_100,Model = "VB", max.age = max(nls_results_dome_100$Estimates$Age), probs = 0.95)


dome_sel_100_plot <- ggplot() +
  geom_point( data = dome_sel_LAA_100, aes(x = Age, y = Lt), alpha = 0.3)+
  labs(y = "Length (cm)", x = "Age (years)")+
  geom_ribbon(data = nls_results_dome_100$Estimates, aes(x = Age, ymin = low, ymax = upp, fill = "Frequentist"),alpha = .5 )+
  geom_line(data = nls_results_dome_100$Estimates, aes(x = Age, y = AVG,col = "Frequentist"),alpha = .5, size = 1.2)+
  geom_lineribbon(data = Bayes_curve_dome_100, aes(x = Age, y = LAA, ymin = .lower, ymax = .upper,fill = "Bayesian",col = "Bayesian"),
                  alpha = .5)+
  geom_line(data = real_curve, aes(x = Age, y = AVG),col = "blue", linetype = "dashed", size = 1.2)+
  scale_x_continuous(expand = c(0,0))+
  scale_y_continuous(expand = c(0,0))+
  scale_fill_viridis_d(end = 0.5, name = "Model")+
  scale_colour_viridis_d(end = 0.5, guide = FALSE)+
  theme(legend.position ="none")

pander(Get_MCMC_parameters(Bayes_results_dome_100),caption = "MCMC - selectivity increases with age")
```

MCMC - selectivity increases with age


| Parameter | mean | se\_mean | sd | 2.5% | 50% | 97.5% | n\_eff | Rhat |
| --- | --- | --- | --- | --- | --- | --- | --- | --- |
| Linf | 257.3 | 0.09 | 7.82 | 243.3 | 256.8 | 274.1 | 7265 | 1 |
| k | 0.19 | 0 | 0.01 | 0.16 | 0.19 | 0.22 | 7461 | 1 |
| L0 | 0.01 | 0 | 0.01 | 0 | 0.01 | 0.02 | 10733 | 1 |
| sigma | 14.75 | 0.01 | 1.06 | 12.86 | 14.69 | 16.97 | 10402 | 1 |

```
pander(rownames_to_column(as.data.frame(nls_results_dome_100$VonB), " "),caption = "nls - selectivity increases with age")
```

nls - selectivity increases with age


|  | Parameter | SE |
| --- | --- | --- |
| Linf | 268.4 | 21.16 |
| k | 0.1494 | 0.04638 |
| L0 | 34.76 | 37.3 |
| RSE | 14.59 | NA |

### Fit models to simulation where n = 150

```
Bayes_results_dome_150 <- Estimate_MCMC_Growth(data = dome_sel_LAA_150,
                                               Model = "VB",
                                               Linf = 275,
                                               k.max = 0.5,
                                               L0 = 0,
                                               L0.se = 0.01,
                                               Linf.se = 27.5,
                                               sigma.max = 50,
                                               BurnIn = 5000,
                                               n_cores = 3,
                                               thin = 100,
                                               iter = 10000)
```

```
nls_results_dome_150 <- Estimate_Growth(data = dome_sel_LAA_150, models = "VB",n.bootstraps = 1000, plots = F,Max.Age = max_age)

Bayes_curve_dome_150 <- Calculate_MCMC_growth_curve(Bayes_results_dome_150,Model = "VB", max.age = max(nls_results_dome_150$Estimates$Age), probs = 0.95)


dome_sel_150_plot <- ggplot() +
  geom_point( data = dome_sel_LAA_150, aes(x = Age, y = Lt), alpha = 0.3)+
  labs(y = "Length (cm)", x = "Age (years)")+
  geom_ribbon(data = nls_results_dome_150$Estimates, aes(x = Age, ymin = low, ymax = upp, fill = "Frequentist"),alpha = .5 )+
  geom_line(data = nls_results_dome_150$Estimates, aes(x = Age, y = AVG,col = "Frequentist"),alpha = .5, size = 1.2)+
  geom_lineribbon(data = Bayes_curve_dome_150, aes(x = Age, y = LAA, ymin = .lower, ymax = .upper,fill = "Bayesian",col = "Bayesian"),
                  alpha = .5)+
  geom_line(data = real_curve, aes(x = Age, y = AVG),col = "blue", linetype = "dashed", size = 1.2)+
  scale_x_continuous(expand = c(0,0))+
  scale_y_continuous(expand = c(0,0))+
  scale_fill_viridis_d(end = 0.5, name = "Model")+
  scale_colour_viridis_d(end = 0.5, guide = FALSE)+
  theme(legend.position ="none")

pander(Get_MCMC_parameters(Bayes_results_dome_150),caption = "MCMC - selectivity increases with age")
```

MCMC - selectivity increases with age


| Parameter | mean | se\_mean | sd | 2.5% | 50% | 97.5% | n\_eff | Rhat |
| --- | --- | --- | --- | --- | --- | --- | --- | --- |
| Linf | 240.3 | 0.06 | 5.27 | 230.7 | 240.1 | 251.4 | 7037 | 1 |
| k | 0.22 | 0 | 0.02 | 0.19 | 0.22 | 0.25 | 7133 | 1 |
| L0 | 0.01 | 0 | 0.01 | 0 | 0.01 | 0.02 | 10857 | 1 |
| sigma | 15.11 | 0.01 | 0.87 | 13.51 | 15.07 | 16.89 | 10961 | 1 |

### Final dome sel plots

```
dome_sel_plots <- plot_grid(dome_sel_cond_plot,dome_sel_50_plot, dome_sel_100_plot, dome_sel_150_plot, align = "h", nrow = 1,labels = c("Dome shaped selectivity", "Sample size = 50", "Sample size = 100", "Sample size = 150"),label_size = 10,label_x = c(-.2,0,0,0))
```

## Missing older individuals

This scenario represents a logistic selectivity function where fishing gear catches young individuals but not older ones. Therefore, Linf is often poorly estimated. The logistic function was set to have an S50 of 1.5 years with a slope of -1. The sample size drawn was 100 individuals.

### Simulate data and plot conditions

```
young_sel_results_50 <- young_selectivity(M = .2, Age = 0:max_age, mean_sel = 1.5, slope_sel = 1,
                                       n = 50, Linf = Linf_obs, k = k_obs , L0 = L0_obs, sigma = sigma_obs)

young_sel_LAA_50 <- young_sel_results_50[[1]] 


young_sel_results_100 <- young_selectivity(M = .2, Age = 0:max_age, mean_sel = 1.5, slope_sel = 1,
                                           n = 100, Linf = Linf_obs, k = k_obs , L0 = L0_obs, sigma = sigma_obs)

young_sel_LAA_100 <- young_sel_results_100[[1]]


young_sel_results_150 <- young_selectivity(M = .2, Age = 0:max_age, mean_sel = 1.5, slope_sel = 1,
                                           n = 150, Linf = Linf_obs, k = k_obs , L0 = L0_obs, sigma = sigma_obs)

young_sel_LAA_150 <- young_sel_results_150[[1]]


young_sel_conditions <-  data.frame(Age = 0:20,
                                    survival = young_sel_results_50[["survival"]] ,
                                    selectivity=  young_sel_results_50[["selectivity"]],
                                    `Relative sampled ages` = young_sel_results_50[["Relative sampled ages"]]) %>%
  set_names(c("Age", "Survival", "Selectivity", "Relative Sampled Ages")) %>%
  pivot_longer(-Age, names_to = "Type",values_to = "Values" )

young_sel_conditions$Type <- factor(young_sel_conditions$Type,levels = c("Survival","Selectivity","Relative Sampled Ages"))


young_sel_cond_plot <- ggplot(young_sel_conditions, aes(x = Age, y = Values, fill = Type, col = Type))+
  ggtitle(" ")+
  geom_area(size = 1, position = position_dodge(), alpha = .5)+
  scale_fill_viridis_d(direction = -1)+
  scale_colour_viridis_d(direction = -1)+
  labs(y = "Proportion", x = "Age (years)")+
  scale_x_continuous(expand = c(0,0))+
  scale_y_continuous(expand = c(0,0))+
  theme_bw()+
  theme(legend.title = element_blank(),
        legend.position = c(0.6,0.8),
        legend.background = element_rect(colour = "black"))
```

### Fit models to simulation where n = 50

```
Bayes_results_young_50 <- Estimate_MCMC_Growth(data = young_sel_LAA_50,
                                          Model = "VB",
                                          Linf = 275,
                                          k.max = 0.5,
                                          L0 = 0,
                                          L0.se = 0.01,
                                          Linf.se = 27.5,
                                          sigma.max = 50,
                                          BurnIn = 5000,
                                          n_cores = 3,
                                          thin = 100,
                                          iter = 10000)
```

```
nls_results_young_50 <- Estimate_Growth(data = young_sel_LAA_50, models = "VB",n.bootstraps = 1000, plots = F,Max.Age = max_age)

Bayes_curve_young_50 <- Calculate_MCMC_growth_curve(Bayes_results_young_50,Model = "VB", max.age = max(nls_results_young_50$Estimates$Age), probs = 0.95)


young_sel_50_plot <- ggplot() +
  geom_point( data = young_sel_LAA_50, aes(x = Age, y = Lt), alpha = 0.3)+
  labs(y = "Length (cm)", x = "Age (years)")+
  geom_ribbon(data = nls_results_young_50$Estimates, aes(x = Age, ymin = low, ymax = upp, fill = "Frequentist"),alpha = .5 )+
  geom_line(data = nls_results_young_50$Estimates, aes(x = Age, y = AVG,col = "Frequentist"),alpha = .5, size = 1.2)+
  geom_lineribbon(data = Bayes_curve_young_50, aes(x = Age, y = LAA, ymin = .lower, ymax = .upper,fill = "Bayesian",col = "Bayesian"),
                  alpha = .5)+
  geom_line(data = real_curve, aes(x = Age, y = AVG),col = "blue", linetype = "dashed", size = 1.2)+
  scale_x_continuous(expand = c(0,0))+
  scale_y_continuous(expand = c(0,0))+
  scale_fill_viridis_d(end = 0.5, name = "Model")+
  scale_colour_viridis_d(end = 0.5, guide = FALSE)+
  theme(legend.position = c(0.25,0.8),
        legend.background = element_rect(colour = "black"))

pander(Get_MCMC_parameters(Bayes_results_young_50),caption = "MCMC - selectivity increases with age")
```

MCMC - selectivity increases with age


| Parameter | mean | se\_mean | sd | 2.5% | 50% | 97.5% | n\_eff | Rhat |
| --- | --- | --- | --- | --- | --- | --- | --- | --- |
| Linf | 269 | 0.29 | 24.22 | 223.8 | 268.2 | 318.9 | 7036 | 1 |
| k | 0.19 | 0 | 0.02 | 0.15 | 0.19 | 0.24 | 6985 | 1 |
| L0 | 0.01 | 0 | 0.01 | 0 | 0.01 | 0.02 | 9631 | 1 |
| sigma | 7.07 | 0.01 | 0.73 | 5.82 | 7 | 8.68 | 10521 | 1 |

### Fit models to simulation where n = 100

```
Bayes_results_young_100 <- Estimate_MCMC_Growth(data = young_sel_LAA_100,
                                          Model = "VB",
                                          Linf = 275,
                                          k.max = 0.5,
                                          L0 = 0,
                                          L0.se = 0.01,
                                          Linf.se = 27.5,
                                          sigma.max = 50,
                                          BurnIn = 5000,
                                          n_cores = 3,
                                          thin = 100,
                                          iter = 10000)
```

```
nls_results_young_100 <- Estimate_Growth(data = young_sel_LAA_100, models = "VB",n.bootstraps = 1000, plots = F,Max.Age = max_age)

Bayes_curve_young_100 <- Calculate_MCMC_growth_curve(Bayes_results_young_100,Model = "VB", max.age = max(nls_results_young_100$Estimates$Age), probs = 0.95)


young_sel_100_plot <- ggplot() +
  geom_point( data = young_sel_LAA_100, aes(x = Age, y = Lt), alpha = 0.3)+
  labs(y = "Length (cm)", x = "Age (years)")+
  geom_ribbon(data = nls_results_young_100$Estimates, aes(x = Age, ymin = low, ymax = upp, fill = "Frequentist"),alpha = .5 )+
  geom_line(data = nls_results_young_100$Estimates, aes(x = Age, y = AVG,col = "Frequentist"),alpha = .5, size = 1.2)+
  geom_lineribbon(data = Bayes_curve_young_100, aes(x = Age, y = LAA, ymin = .lower, ymax = .upper,fill = "Bayesian",col = "Bayesian"),
                  alpha = .5)+
  geom_line(data = real_curve, aes(x = Age, y = AVG),col = "blue", linetype = "dashed", size = 1.2)+
  scale_x_continuous(expand = c(0,0))+
  scale_y_continuous(expand = c(0,0))+
  scale_fill_viridis_d(end = 0.5, name = "Model")+
  scale_colour_viridis_d(end = 0.5, guide = FALSE)+
  theme(legend.position ="none")

pander(Get_MCMC_parameters(Bayes_results_young_100),caption = "MCMC - selectivity increases with age")
```

MCMC - selectivity increases with age


| Parameter | mean | se\_mean | sd | 2.5% | 50% | 97.5% | n\_eff | Rhat |
| --- | --- | --- | --- | --- | --- | --- | --- | --- |
| Linf | 271.4 | 0.28 | 22.38 | 229.4 | 270.8 | 316.4 | 6509 | 1 |
| k | 0.18 | 0 | 0.02 | 0.15 | 0.18 | 0.23 | 6519 | 1 |
| L0 | 0.01 | 0 | 0.01 | 0 | 0.01 | 0.02 | 9319 | 1 |
| sigma | 6.56 | 0 | 0.47 | 5.71 | 6.53 | 7.55 | 11108 | 1 |

### Fit models to simulation where n = 150

```
Bayes_results_young_150 <- Estimate_MCMC_Growth(data = young_sel_LAA_150,
                                          Model = "VB",
                                          Linf = 275,
                                          k.max = 0.5,
                                          L0 = 0,
                                          L0.se = 0.01,
                                          Linf.se = 27.5,
                                          sigma.max = 50,
                                          BurnIn = 5000,
                                          n_cores = 3,
                                          thin = 100,
                                          iter = 10000)
```

```
nls_results_young_150 <- Estimate_Growth(data = young_sel_LAA_150, models = "VB",n.bootstraps = 1000, plots = F,Max.Age = max_age)

Bayes_curve_young_150 <- Calculate_MCMC_growth_curve(Bayes_results_young_150,Model = "VB", max.age = max(nls_results_young_150$Estimates$Age), probs = 0.95)


young_sel_150_plot <- ggplot() +
  geom_point( data = young_sel_LAA_150, aes(x = Age, y = Lt), alpha = 0.3)+
  labs(y = "Length (cm)", x = "Age (years)")+
  geom_ribbon(data = nls_results_young_150$Estimates, aes(x = Age, ymin = low, ymax = upp, fill = "Frequentist"),alpha = .5 )+
  geom_line(data = nls_results_young_150$Estimates, aes(x = Age, y = AVG,col = "Frequentist"),alpha = .5, size = 1.2)+
  geom_lineribbon(data = Bayes_curve_young_150, aes(x = Age, y = LAA, ymin = .lower, ymax = .upper,fill = "Bayesian",col = "Bayesian"),
                  alpha = .5)+
  geom_line(data = real_curve, aes(x = Age, y = AVG),col = "blue", linetype = "dashed", size = 1.2)+
  scale_x_continuous(expand = c(0,0))+
  scale_y_continuous(expand = c(0,0))+
  scale_fill_viridis_d(end = 0.5, name = "Model")+
  scale_colour_viridis_d(end = 0.5, guide = FALSE)+
  theme(legend.position ="none")

pander(Get_MCMC_parameters(Bayes_results_young_150),caption = "MCMC - selectivity increases with age")
```

MCMC - selectivity increases with age


| Parameter | mean | se\_mean | sd | 2.5% | 50% | 97.5% | n\_eff | Rhat |
| --- | --- | --- | --- | --- | --- | --- | --- | --- |
| Linf | 278.2 | 0.26 | 21.3 | 238.5 | 277.7 | 321.6 | 6466 | 1 |
| k | 0.18 | 0 | 0.02 | 0.15 | 0.18 | 0.22 | 6490 | 1 |
| L0 | 0.01 | 0 | 0.01 | 0 | 0.01 | 0.02 | 10953 | 1 |
| sigma | 6.59 | 0 | 0.38 | 5.89 | 6.58 | 7.4 | 10520 | 1 |

```
pander(rownames_to_column(as.data.frame(nls_results_young_150$VonB), " "),caption = "nls - selectivity increases with age")
```

nls - selectivity increases with age


|  | Parameter | SE |
| --- | --- | --- |
| Linf | 285.9 | 38.82 |
| k | 0.1722 | 0.02898 |
| L0 | 0.08225 | 0.8007 |
| RSE | 6.575 | NA |

### Final young sel plots

```
young_sel_plots <- plot_grid(young_sel_cond_plot,young_sel_50_plot, young_sel_100_plot, young_sel_150_plot, align = "h", nrow = 1,labels = c("Selectivity decreases with age", "Sample size = 50", "Sample size = 100", "Sample size = 150"),label_size = 10,label_x = c(-.2,0,0,0))
```

## All plots

Print and Save final figure for the manuscript

```
Main_figure <- plot_grid(dome_sel_plots, young_sel_plots, Old_sel_plots, nrow = 3)
Main_figure
```
