# Supplementary material for: Modernising fish and shark growth curves with Bayesian length-at-age models
Source: PLoS One. 2021 Feb 8;16(2):e0246734. doi: 10.1371/journal.pone.0246734 (PMC7870076; doi:10.1371/journal.pone.0246734)
Supplement: S3 Appendix — (HTML) [file pone.0246734.s003.html]

Appendix 3: Model comparison using different standard errors for priors


# Appendix 3: Model comparison using different standard errors for priors

## Load libaries

```
library(tidyverse)
library(truncnorm)
library(AquaticLifeHistory)
library(BayesGrowth)
library(tidybayes)
library(bayesplot)
library(tmvtnorm)
library(cowplot)
library(pander)

theme_set(theme_bw())
```

# Narrow vs wide priors for L0 and Linf

## Silvertip sharks

Run MCMC models using base case priors, where the standard error of each prior is 25% of the point estimate and where the standard error of each prior is 50% of the point estimate.

```
prior_base_model_ALS <- Estimate_MCMC_Growth(silvertips,
                                Model = "VB" ,
                                iter = 10000,
                                n.chains = 4,
                                BurnIn = 5000,
                                thin = 1,
                                Linf = 300,
                                Linf.se = 30,
                                L0 = 70,
                                L0.se = 7,
                                sigma.max = 100,
                                verbose = T,
                                n_cores = 3,
                                k.max = 0.3)

prior_25_model_ALS <- Estimate_MCMC_Growth(silvertips,
                                Model = "VB" ,
                                iter = 10000,
                                n.chains = 4,
                                BurnIn = 5000,
                                thin = 1,
                                Linf = 300,
                                Linf.se = 75,
                                L0 = 68,
                                L0.se = 17.5,
                                sigma.max = 100,
                                verbose = T,
                                n_cores = 3,
                                k.max = 0.3)


prior_50_model_ALS <- Estimate_MCMC_Growth(silvertips,
                                Model = "VB" ,
                                iter = 10000,
                                n.chains = 4,
                                BurnIn = 5000,
                                thin = 1,
                                Linf = 300,
                                Linf.se = 150,
                                L0 = 68,
                                L0.se = 35,
                                sigma.max = 100,
                                verbose = T,
                                n_cores = 3,
                                k.max = 0.3)
```

Plot the growth curves and print the results.

```
prior_base_curve_ALS <- Calculate_MCMC_growth_curve(prior_base_model_ALS,Model = "VB", max.age = 35, probs = 0.95)
prior_25_curve_ALS <- Calculate_MCMC_growth_curve(prior_25_model_ALS,Model = "VB", max.age = 35, probs = 0.95)
prior_50_curve_ALS <- Calculate_MCMC_growth_curve(prior_50_model_ALS,Model = "VB", max.age = 35, probs = 0.95)


Prior_comp_plot_ALS <- ggplot() +
  ggtitle("Silvertip Shark")+
  geom_point( data = silvertips, aes(x = Age, y = Length), alpha = 0.3)+
  labs(y = "Length (cm)", x = "Age (years)")+
  geom_lineribbon(data = prior_base_curve_ALS, aes(x = Age, y = LAA, ymin = .lower, ymax = .upper,fill = "Base SE of the Prior",col = "Base SE of the Prior"),
                  alpha = .4)+
  geom_lineribbon(data = prior_25_curve_ALS, aes(x = Age, y = LAA, ymin = .lower, ymax = .upper,fill = "SE 25% is of the Prior",col = "SE 25% is of the Prior"),
                  alpha = .4)+
  geom_lineribbon(data = prior_50_curve_ALS, aes(x = Age, y = LAA, ymin = .lower, ymax = .upper,fill = "SE 50% is of the Prior",col = "SE 50% is of the Prior"),
                  alpha = .4)+
  geom_line(data = prior_base_curve_ALS, aes(x = Age, y = LAA,col = "Base SE of the Prior"),size = 1)+
  geom_line(data = prior_25_curve_ALS, aes(x = Age, y = LAA,col = "SE 25% is of the Prior"),size = 1)+
  geom_line(data = prior_50_curve_ALS, aes(x = Age, y = LAA,col = "SE 50% is of the Prior"),size = 1)+
  expand_limits(y = 0)+
  scale_x_continuous(expand = c(0,0))+
  scale_y_continuous(expand = c(0,0))+
  scale_fill_viridis_d(direction = -1,name = NULL)+
  scale_colour_viridis_d(direction = -1,guide = F)+
  theme(legend.position = c(0.8,.2),
        legend.background = element_rect(colour = "black"))

Prior_comp_plot_ALS
```

Print a table of each result.

```
pander(Get_MCMC_parameters(prior_base_model_ALS),caption = "Base SE of the Prior")
```

Base SE of the Prior


| Parameter | mean | se\_mean | sd | 2.5% | 50% | 97.5% | n\_eff | Rhat |
| --- | --- | --- | --- | --- | --- | --- | --- | --- |
| Linf | 296.6 | 0.22 | 22.4 | 257.1 | 295.3 | 344.6 | 10556 | 1 |
| k | 0.06 | 0 | 0.01 | 0.04 | 0.06 | 0.08 | 10323 | 1 |
| L0 | 77.36 | 0.04 | 4.24 | 68.77 | 77.43 | 85.45 | 14279 | 1 |
| sigma | 11.69 | 0.01 | 1.32 | 9.44 | 11.58 | 14.61 | 15291 | 1 |

```
pander(Get_MCMC_parameters(prior_25_model_ALS),caption = "SE 25% is of the Prior")
```

SE 25% is of the Prior


| Parameter | mean | se\_mean | sd | 2.5% | 50% | 97.5% | n\_eff | Rhat |
| --- | --- | --- | --- | --- | --- | --- | --- | --- |
| Linf | 356.4 | 0.47 | 47.77 | 277.4 | 351.6 | 462.6 | 10400 | 1 |
| k | 0.04 | 0 | 0.01 | 0.02 | 0.04 | 0.06 | 9967 | 1 |
| L0 | 90.97 | 0.05 | 5.36 | 79.62 | 91.24 | 100.8 | 12951 | 1 |
| sigma | 10.82 | 0.01 | 1.2 | 8.76 | 10.72 | 13.46 | 15531 | 1 |

```
pander(Get_MCMC_parameters(prior_50_model_ALS),caption = "SE 50% is of the Prior")
```

SE 50% is of the Prior


| Parameter | mean | se\_mean | sd | 2.5% | 50% | 97.5% | n\_eff | Rhat |
| --- | --- | --- | --- | --- | --- | --- | --- | --- |
| Linf | 419.4 | 0.91 | 85.08 | 291.4 | 406.2 | 618.6 | 8684 | 1 |
| k | 0.03 | 0 | 0.01 | 0.02 | 0.03 | 0.06 | 8063 | 1 |
| L0 | 94.44 | 0.05 | 5.27 | 83.21 | 94.74 | 104 | 10503 | 1 |
| sigma | 10.64 | 0.01 | 1.16 | 8.66 | 10.54 | 13.21 | 14501 | 1 |

## Silky sharks

Run MCMC models using base case priors, where the standard error of each prior is 25% of the point estimate and where the standard error of each prior is 50% of the point estimate.

```
prior_base_model_FAL <- Estimate_MCMC_Growth(silky,
                                Model = "Log" ,
                                iter = 10000,
                                n.chains = 4,
                                BurnIn = 5000,
                                thin = 1,
                                Linf = 280,
                                Linf.se = 15,
                                L0 = 77,
                                L0.se = 5,
                                sigma.max = 100,
                                verbose = T,
                                n_cores = 3,
                                k.max = 5)

prior_25_model_FAL <- Estimate_MCMC_Growth(silky,
                                Model = "Log" ,
                                iter = 10000,
                                n.chains = 4,
                                BurnIn = 5000,
                                thin = 1,
                                Linf = 280,
                                Linf.se = 70,
                                L0 = 77,
                                L0.se = 19.25,
                                sigma.max = 100,
                                verbose = T,
                                n_cores = 3,
                                k.max = 5)


prior_50_model_FAL <- Estimate_MCMC_Growth(silky,
                                Model = "Log" ,
                                iter = 10000,
                                n.chains = 4,
                                BurnIn = 5000,
                                thin = 1,
                                Linf = 280,
                                Linf.se = 140,
                                L0 = 77,
                                L0.se = 15.4,
                                sigma.max = 100,
                                verbose = T,
                                n_cores = 3,
                                k.max = 5)
```

Plot the growth curves and print the results.

```
prior_base_curve_FAL <- Calculate_MCMC_growth_curve(prior_base_model_FAL,Model = "Log",max.age = 27, probs = c(.95))
prior_25_curve_FAL <- Calculate_MCMC_growth_curve(prior_25_model_FAL,Model = "Log",max.age = 27, probs = c(.95))
prior_50_curve_FAL <- Calculate_MCMC_growth_curve(prior_50_model_FAL,Model = "Log",max.age = 27, probs = c(.95))


Prior_comp_plot_FAL <- ggplot() +
  ggtitle("Silky Shark")+
  geom_point( data = silky, aes(x = Age, y = Length), alpha = 0.3)+
  labs(y = "Length (cm)", x = "Age (years)")+
  geom_lineribbon(data = prior_base_curve_FAL, aes(x = Age, y = LAA, ymin = .lower, ymax = .upper,fill = "Base SE of the Prior",col = "Base SE of the Prior"),
                  alpha = .4)+
  geom_lineribbon(data = prior_25_curve_FAL, aes(x = Age, y = LAA, ymin = .lower, ymax = .upper,fill = "SE 25% is of the Prior",col = "SE 25% is of the Prior"),
                  alpha = .4)+
  geom_lineribbon(data = prior_50_curve_FAL, aes(x = Age, y = LAA, ymin = .lower, ymax = .upper,fill = "SE 50% is of the Prior",col = "SE 50% is of the Prior"),
                  alpha = .4)+
  geom_line(data = prior_base_curve_FAL, aes(x = Age, y = LAA,col = "Base SE of the Prior"),size = 1)+
  geom_line(data = prior_25_curve_FAL, aes(x = Age, y = LAA,col = "SE 25% is of the Prior"),size = 1)+
  geom_line(data = prior_50_curve_FAL, aes(x = Age, y = LAA,col = "SE 50% is of the Prior"),size = 1)+
  expand_limits(y = 0)+
  scale_x_continuous(expand = c(0,0))+
  scale_y_continuous(expand = c(0,0))+
  scale_fill_viridis_d(direction = -1,name = NULL)+
  scale_colour_viridis_d(direction = -1,guide = F)+
  theme(legend.position = "none")

Prior_comp_plot_FAL
```

Print a table of each result.

```
pander(Get_MCMC_parameters(prior_base_model_FAL),caption = "Base SE of the Prior")
```

Base SE of the Prior


| Parameter | mean | se\_mean | sd | 2.5% | 50% | 97.5% | n\_eff | Rhat |
| --- | --- | --- | --- | --- | --- | --- | --- | --- |
| Linf | 269 | 0.06 | 5.36 | 259.1 | 268.8 | 280 | 6978 | 1 |
| k | 0.14 | 0 | 0.01 | 0.13 | 0.14 | 0.15 | 6164 | 1 |
| L0 | 82.31 | 0.02 | 1.57 | 79.21 | 82.32 | 85.39 | 7281 | 1 |
| sigma | 14.67 | 0 | 0.45 | 13.81 | 14.65 | 15.59 | 10281 | 1 |

```
pander(Get_MCMC_parameters(prior_25_model_FAL),caption = "SE 25% is of the Prior")
```

SE 25% is of the Prior


| Parameter | mean | se\_mean | sd | 2.5% | 50% | 97.5% | n\_eff | Rhat |
| --- | --- | --- | --- | --- | --- | --- | --- | --- |
| Linf | 268.9 | 0.07 | 5.81 | 258.4 | 268.6 | 281.3 | 6676 | 1 |
| k | 0.14 | 0 | 0.01 | 0.12 | 0.14 | 0.15 | 6142 | 1 |
| L0 | 82.63 | 0.02 | 1.67 | 79.39 | 82.62 | 85.95 | 7270 | 1 |
| sigma | 14.67 | 0 | 0.46 | 13.8 | 14.66 | 15.61 | 9994 | 1 |

```
pander(Get_MCMC_parameters(prior_50_model_FAL),caption = "SE 50% is of the Prior")
```

SE 50% is of the Prior


| Parameter | mean | se\_mean | sd | 2.5% | 50% | 97.5% | n\_eff | Rhat |
| --- | --- | --- | --- | --- | --- | --- | --- | --- |
| Linf | 268.8 | 0.07 | 5.87 | 258.1 | 268.5 | 281.1 | 6888 | 1 |
| k | 0.14 | 0 | 0.01 | 0.12 | 0.14 | 0.15 | 6329 | 1 |
| L0 | 82.64 | 0.02 | 1.68 | 79.33 | 82.63 | 85.93 | 7533 | 1 |
| sigma | 14.67 | 0 | 0.46 | 13.8 | 14.65 | 15.62 | 9062 | 1 |

## Final plot

```
Prior_plots <- plot_grid(Prior_comp_plot_ALS, Prior_comp_plot_FAL, nrow = 1)
Prior_plots
```
